# Pharmacologic stimulation of insulin granule acidification increases β-cell zinc content and augments β-cell-targeted drug delivery

**DOI:** 10.1016/j.jbc.2025.110645

**Published:** 2025-08-28

**Authors:** Sooyeon Lee, Hannah P. Fraser, Rebecca C. Schugar, Haixia Xu, Timothy M. Horton, Ella A. Thomson, Julie Park, Xucheng Zhang, Justin P. Annes

**Affiliations:** 1 Division of Endocrinology, Department of Medicine, Stanford University, Stanford, California, USA; 2 Department of Electrical Engineering, Stanford University, Stanford, California, USA; 3 Department of Bioengineering, Stanford University, Stanford, California, USA; 4 Stanford ChEM-H and Diabetes Research Center, Stanford University School of Medicine, Stanford, California, USA

**Keywords:** beta cell (β-cell), pancreatic islet, zinc, drug delivery, β-cell replication, ZnT8, V-ATPase, cAMP, PKA, vesicle acidification

## Abstract

Pathologic loss of insulin-producing pancreatic β-cells is a hallmark of diabetes that is potentially reversible through regenerative therapy. However, existing replication-promoting compounds lack β-cell specificity, limiting their clinical application. To overcome this challenge, we generated βRepZnC, a zinc-chelating replication compound designed to leverage the uniquely high zinc content of β-cells for targeted delivery. Herein, we identify pharmacological agents that boost β-cell zinc and improve the targeted delivery and bioactivity of βRepZnC. Using a high-content, image-based screen with the zinc fluorophore TSQ, we identified GR-46611 as a pharmacologic enhancer of β-cell zinc levels. Time-lapse TSQ imaging revealed that GR-46611 rapidly elevated intracellular zinc, prompting further mechanistic studies that showed increased zinc accumulation through the transporter ZnT8. This effect was mediated by enhanced V-ATPase–driven vesicle acidification *via* cAMP–PKA signaling inhibition. Supporting this mechanism, multiple protein kinase A (PKA) inhibitors also increased β-cell zinc content. Importantly, zinc enhancement significantly increased βRepZnC accumulation in both mouse and human primary islets, with fluorescence-activated cell sorting and mass spectrometry confirming selective drug retention in β-cells over non-β-cells. To evaluate effects on bioactivity, we performed complementary on-treatment and post treatment islet replication assays, measuring replication either concurrent with or 48 h after drug exposure, respectively. Zinc elevation *via* GR-46611 or the PKA inhibitor H89 selectively potentiated βRepZnC-induced β-cell replication in both contexts. Notably, only βRepZnC—unlike non-zinc-binding replication compounds—elicited a sustained replication response after drug withdrawal. This work defines a new pharmacologic strategy for manipulating β-cell zinc levels that can be exploited for durable β-cell-targeted therapeutic delivery.

Diabetes is the most common human endocrine disease in the world, affecting over 422 million adults worldwide (https://www.who.int/news-room/fact-sheets/detail/diabetes). Although different pathogenic mechanisms cause type 1 diabetes (T1D) and type 2 diabetes (T2D), loss of insulin-producing β-cells is a contributing feature of both (1, 2). A variety of strategies for replacing or expanding an individual’s β-cell mass are being pursued for therapeutic development. Developing a regenerative therapy that can expand pancreatic β-cell mass, boost endogenous insulin production, and restore glucose homeostasis presents a transformative opportunity for diabetes treatment, where β-cell loss is a contributing factor to disease progression (1, 2, 3). However, the development of improved diabetes medications is stymied by a dearth of safe therapeutic targets. On-target but off-tissue drug effects are slowing progress across multiple therapeutic domains including β-cell regeneration, β-cell preservation, and immune-protection (4). In principle, stimulating the regeneration of insulin-producing β-cells could be used to restore endogenous insulin production capacity (5, 6, 7).

Mature human β-cells exhibit limited native capacity for expansion and are relatively resistant to replication (8). Nevertheless, medicinal chemistry efforts have yielded several compounds that potently induce the replication of quiescent mature β-cells by inhibition of the enzyme dual-specificity tyrosine phosphorylation-regulated kinase 1A (DYRK1A), including 5-iodotubericidin, harmine, CC-401, GNF-4877, and GNF-2133 (5, 6, 7, 9, 10, 11, 12, 13, 14, 15). DYRK1A plays a central role in maintaining quiescence in various cell types by suppressing NFAT activity (6, 10, 11), stabilizing p27kip1 (9, 12) and maintaining DREAM-complex target gene repression (9, 16, 17). Thus, one of the major challenges hindering clinical translation rests in the development of methodologies for β-cell-selective drug delivery to minimize off-target growth effects on non-β cells (3). Consequently, a “modular” system for β-cell-targeted drug delivery may help to realize the next generation of diabetes therapeutics.

To address this challenge, we and others developed β-cell-targeted drug delivery modules that leverage the extraordinarily high zinc content of β-cells (18, 19, 20). β-cells uniquely concentrate zinc in insulin vesicles to levels ranging from [10–20 mM] (21), substantially higher than α-cells (22, 23) and other cell types [400 pM-300 μM] (24). In β-cell granules, zinc is essential for insulin crystallization—each insulin hexamer incorporates ∼12 zinc ions—and contributes to the formation of the mature dense core granules characteristic of β-cells (25). All zinc transporter (ZnT; *SLC30*) and Zrt-/Irt-like protein (ZIP; *SLC39*) family members are expressed in pancreatic islets (26, 27), underscoring the importance of zinc homeostasis in β-cell function. Among these, ZnT8 (*SLC30A8*) plays a dominant role in transporting zinc into insulin granules and is uniquely enriched in β-cells with a highly restricted expression pattern (28, 29, 30).

Primarily based upon structural data, ZnT8 is proposed to act as a zinc-H^+^ ion antiporter where the transport of zinc into the granule lumen is controlled by the counter-transport of two protons and granular release of zinc is driven by H^+^ displacement (31, 32). However, the precise mechanism by which ZnT8 senses and responds to vesicle acidification to facilitate zinc import remains incompletely understood. This gap in knowledge is particularly relevant given that ZnT8-mediated zinc transport is essential for granule zinc loading: ZnT8 knockout markedly reduces β-cell zinc content (33, 34, 35), while ZnT8 overexpression enhances zinc accumulation (36, 37). Furthermore, genetic studies in humans have highlighted the clinical significance of ZnT8: loss-of-function mutations, including the p.Arg138∗ truncating variant, are associated with a ∼50% reduced risk of developing type 2 diabetes, whereas systemic or dietary zinc depletion has been linked to β-cell dysfunction and diabetes (38). These findings underscore the importance of defining the molecular and signaling mechanisms that regulate ZnT8 activity and β-cell zinc homeostasis. A deeper understanding of these pathways—particularly how acidification and transporter function are coupled—may offer new insights into β-cell biology and open avenues for therapeutic intervention in diabetes.

In our β-cell-targeted drug delivery system, a zinc-chelating moiety is covalently linked to the replication-promoting compound, GNF-4877 to generate βRepZnC, which is bioconcentrated within β-cells by the zinc-binding moiety and preferentially induces β-cell replication *via* DYRK1A inhibition (18). Similarly targeting zinc-rich β-cells, others have designed a zinc-binding prodrug (ZnPD) that utilizes a zinc-catalyzed release of replication compounds, like GNF-4877 (19) or harmine (20). Although these zinc-binding prodrugs were shown to enhance human β-cell replication, their β-cell selectivity remains unclear. In this study, we performed chemical screening on β-cells to identify pharmacological agents that boost β-cell zinc content, enhancing the potential applicability of βRepZnCs and uncovering fundamental aspects of β-cell zinc regulation.

## Results

### Chemical screening identifies GR-46611 as a pharmacological enhancer of β-cell zinc content

**Figure 1:**
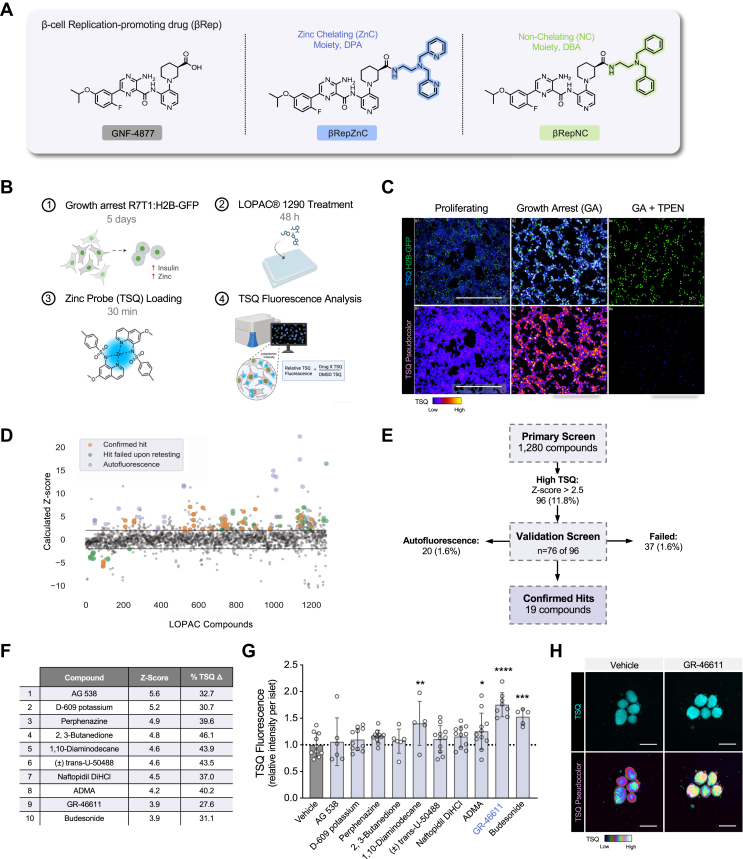
**Chemical screening identifies GR-46611 as an enhancer of β-cell zinc content.***A*, structures of β-cell replication-promoting (βRep) drugs: GNF-4877 and hybrid molecules, βRepZnC and βRepNC, generated by attaching a zinc-chelating moiety (2,2′-dipicoylamine; DPA) or nonchelating isostere (dibenzylamine; DBA) to the GNF-4877 carboxylate, respectively. *B*, overview of TSQ-based chemical screening protocol: (1) R7T1:H2B-GFP β-cells were growth arrested for 5 days to maximize insulin and zinc content of β-cells prior to screening; (2) cells were plated and treated with 1 μM or 10 μM compound for 48 h (*n* = 2/compound); (3) cells were exposed to 100 μM TSQ for 30 min to probe zinc; and (4) TSQ fluorescence intensity (relative to DMSO-treated wells) was determined for each compound. Created with BioRender.com. *C*, representative images of TSQ staining in proliferating and growth arrested R7T1:H2B-GFP β-cells. TSQ fluorescence shown in *blue* (*top*) and by pseudocolor spectrum (*bottom*) to enhance visualization. Addition of 100 μM TPEN prevented TSQ fluorescence. *D*, calculated Z-scores for 1280 compounds based on TSQ fluorescence. Compounds confirmed (*orange*) or rejected (*green* and *lavender*) in follow-up testing are highlighted (*n* = 7/compound). *E*, summary of screening results identifying β-cell zinc enhancers. *F*, top 10 β-cell zinc enhancing compounds based on Z-score and % change in TSQ fluorescence relative to vehicle control. *G*, TSQ fluorescence analysis of top 10 hit compounds in intact mouse islets. Data represent relative TSQ intensity per islet (*n* = 5–11/compound). *H*, live-fluorescence imaging of TSQ-stained mouse islets following vehicle or 10 μM GR-46611 treatment for 24 h. The scale bars represent 200 μm. Data represent mean ± SD. One-way ANOVA with Fisher’s LSD *post hoc* test for (*G*). ∗∗∗∗*p* < 0.0001, ∗∗∗*p* < 0.001, ∗∗*p* < 0.01, ∗*p* < 0.05. DMSO, dimethyl sulfoxide; LSD, least significant difference; TPEN, N,N,N′,N′-tetrakis(2-pyridylmethyl)-1,2-ethanediamine; TSQ, 6-methoxy-8-p-toluenesulfonamido-quinoline.

Previously, we generated a zinc-chelating derivative of GNF-4877, designed to target drug delivery and bioactivity toward zinc-rich β-cells (18). This compound, βRepZnC, was generated by attaching a zinc-chelating moiety 2,2′-dipicoylamine (DPA) to the GNF-4877 carboxylate with a spacer (Fig. 1*A*). As a control, a nonchelating version, βRepNC, was generated by similarly integrating a nonchelating moiety, dibenzylamine (DBA) into GNF-4877 (Fig. 1*A*). Here, we pursued a two-pronged strategy: first, to identify a pharmacological agent that acutely enhances intracellular zinc levels in β-cells; and second, to test whether this zinc-enhancing treatment potentiates βRepZnC delivery and its downstream effect on β-cell replication. This approach allowed us to directly test whether pharmacological elevation of β-cell zinc improves β-cell–specific delivery and functional efficacy.

To identify pharmacological agents that enhance β-cell zinc content, we screened ∼1280 bioactive compounds using reversibly transformed mouse R7T1 β-cells engineered to express nuclear H2B-GFP (R7T1:H2B-GFP β-cells) for efficient live-cell imaging analysis (Fig. 1*B*). For screening, R7T1:H2B-GFP β-cells were growth arrested by doxycycline withdrawal (5 days) to enhance insulin granule content (39) and emulate the zinc-rich phenotype of healthy human β-cells (Fig. 1, *B* and *C*). Zinc content was assessed using (6-methoxy-8-p-toluenesulfonamido-quinoline (TSQ), a membrane-permeable fluorescent turn-on zinc probe well-suited for detecting physiologic changes in β-cell zinc content (40, 41, 42). The dependence of TSQ fluorescence on zinc was confirmed by its quenching with excess zinc chelator, N,N,N′,N′-tetrakis(2-pyridylmethyl)-1,2-ethanediamine (TPEN) (Fig. 1*C*). For chemical screening, TSQ fluorescence intensity was quantified in the cytoplasm (excluding the nuclei) of compound-treated cells. This system provided a robust platform for identifying zinc-modulating agents.

Hit compounds were identified by a significant increase or decrease (Z-score >|2.5|) in TSQ fluorescence intensity relative to vehicle-treated β-cells (Fig. 1*D*). Of the 1280 compounds, 96 compounds increased fluorescence, and 55 compounds decreased fluorescence. Next, we selected 76 of the 96 zinc-increasing hit compounds for autofluorescence assessment (compound treatment without TSQ) and for evaluation in higher replicate numbers (Fig. 1*E*). Ultimately, retesting confirmed 19 compounds that increased TSQ fluorescence in R7T1:H2B-GFP β-cells (Table S1). The top 10 confirmed hits (Z-scores ≥3.9) were further tested in primary mouse islets using live-cell TSQ staining (Fig. 1, *F* and *G*). Of these, three compounds (budesonide, 1,10-diaminodecane and GR-46611)- significantly increased islet TSQ fluorescence (Fig. 1*G*), with GR-46611 showing the strongest effect (Fig. 1*H*). Consequently, subsequent experiments focused on GR-46611, which was generally used at 10 μM due to its robust activity and tolerability (Fig. S1).

### GR-46611 enhances βRepZnC accumulation in islets

**Figure 2:**
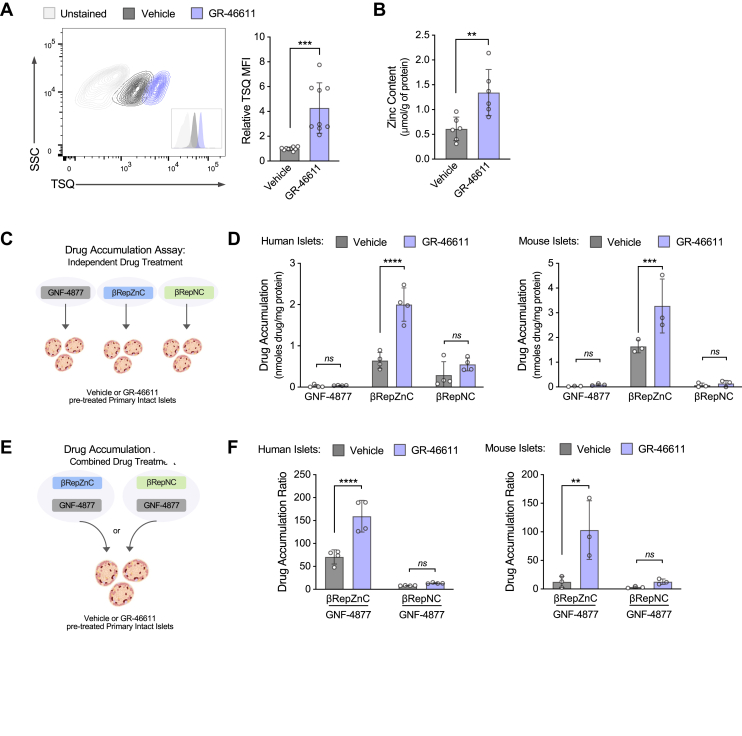
**GR-46611 increases human and mouse islet accumulation of βRepZnC.***A*, representative FACS contour plots of TSQ analysis in mouse islets treated with vehicle or GR-46611 treatment for 24 h. Data represent mean TSQ MFI relative to vehicle (*n* = 9). *B*, human islet zinc content following vehicle or GR-46611 treatment for 24 h (*n* = 4 replicates from one donor). *C*, schematic representation of drug accumulation measurements in islets pretreated with vehicle or 10 μM GR-46611 followed by independent treatment with 1.5 μM βRep drugs for 72 h. *D*, βRep drug accumulation measurements in human (*n* = 4 replicates from one donor) and mouse (*n* = 3) islets following independent treatment. *E*, schematic representation of the drug accumulation assay in measurements in islets pretreated with vehicle or GR-46611 followed by combined treatments of either βRepZnC or βRepNC with GNF-4877 for 72 h. *F*, drug accumulation ratio measured in human (*n* = 4 replicates from one donor) and mouse (*n* = 3) islets following combined treatments. The scale bars represent 100 μm. Data represent mean ± SD. Unpaired Student’s *t* test assuming Gaussian distribution for (*A* and *B*); two-way ANOVA with Fisher’s LSD *post hoc* test for (*D* and *F*); ∗∗∗∗*p* < 0.0001, ∗∗∗*p* < 0.001, ∗∗*p* < 0.01, *ns*, nonsignificant. FACS, fluorescence-activated cell sorting; LSD, least significant difference; MFI, mean fluorescence intensity; TSQ, 6-methoxy-8-p-toluenesulfonamido-quinoline.

To validate the effects of GR-46611 on β-cell zinc content, we measured TSQ fluorescence in primary mouse islet β-cells by fluorescence-activated cell sorting (FACS) as a complimentary methodology (Fig. 2*A*). Indeed, GR-46611-treatment caused a ∼4-fold increase in mean TSQ fluorescence, relative to vehicle-treated islets. Next, we directly measured zinc content in human cadaveric islets using inductively coupled plasma-optical emission spectrometry. Consistent with TSQ-based findings, GR-46611 treatment increased intracellular zinc by ∼2-fold in human islets (Fig. 2*B*). Hence, we identified GR-46611 as, to our knowledge, the first chemical enhancer of mature β-cell zinc content.

Next, we developed a primary islet-based assay to determine whether GR-46611 enhanced islet βRepZnC accumulation (Fig. 2*C*). Isolated mouse or human islets were pretreated with vehicle or GR-46611, followed by the addition of β-cell replication compounds (βRep: GNF-4877, βRepZnC, or βRepNC; 72 h). Compound accumulation in islets was then measured by liquid chromatography–tandem mass spectrometry. Consistent with our previous findings (18), vehicle-treated islets accumulated βRepZnC at significantly higher concentrations than GNF-4877 and βRepNC (Fig. 2*D*). Remarkably, GR-46611 treatment increased βRepZnC accumulation by 2- to 3-fold compared to vehicle-treated islets, resulting in an impressive 52-fold increased βRepZnC in human islets and a 35-fold increase in mouse islets relative to the parent molecule GNF-4877 (Fig. 2*D*). Next, we modified the islet-based drug accumulation assay to include cotreatment with GNF-4877 in all conditions, providing an internal control for normalization (Fig. 2*E*). Normalized βRepZnC accumulation was significantly higher in both human and mouse islets of GR-46611-treated islets compared to vehicle-treated islets (Fig. 2*F*). The substantial improvement in βRepZnC islet accumulation, relative to GNF-4877, was confirmed in both human (71-fold) and mouse (13-fold) islets (Fig. 2*F*). Strikingly, GR-46611 treatment further increased islet accumulation of βRepZnC accumulation to 160-fold in human islets and 103-fold in mouse islets (relative to GNF-4877) but had no effect on islet βRepNC accumulation (Fig. 2*F*), demonstrating GR-46611’s specific impact on βRepZnC accumulation in islets. Collectively, these data indicated that GR-46611-induced islet zinc content led to increased islet accumulation of βRepZnC, but not βRepNC.

### GR-46611 augments β-cell-targeted βRepZnC delivery by selectively inducing zinc accumulation in β-cells

**Figure 3:**
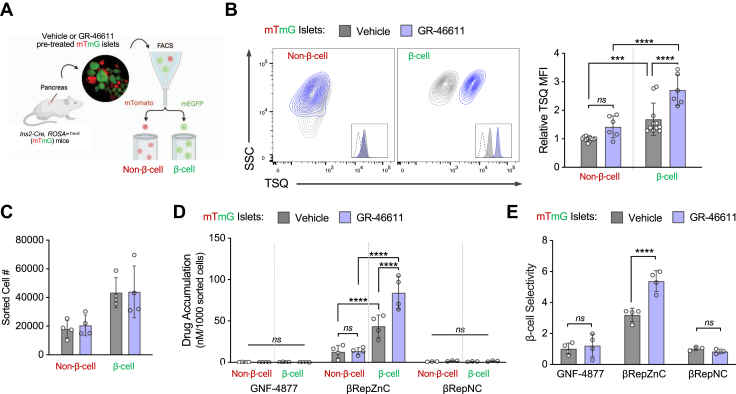
**GR-46611 selectively increases β-cell zinc content and enhances β-cell-targeted drug delivery.***A*, schematic representation of the experimental strategy used to independently analyze islet non-β-cells (*red*) and β-cells (*green*) obtained from *Ins2-Cre ROSA*^*mT/mG*^ (mTmG) mice. *B*, representative FACS contour plots of TSQ analysis in non-β-cells (*red*) and β-cells (*green*) obtained from mTmG mouse islets treated with vehicle or GR-46611 treatment for 24 h. Data represent mean TSQ MFI relative to vehicle-treated β-cells (*n* = 6–11 mice). Histogram includes unstained sample as *dotted line*. *C*, number of non-β-cells and β-cells sorted from vehicle- or GR-46611-treated mTmG islets (*n* = islets pooled from eight mice). *D*, βRep drug accumulation measurements in non-β-cells and β-cells sorted from *mTmG* islets pretreated with vehicle or GR-46611 (*n* = 4 mice). Data represent mean concentration normalized to cell number. *E*, β-cell selectivity of βRep drug accumulation calculated by the concentration in β-cells/non-β-cells. The scale bars represent 200 μm. Data represent mean ± SD. Two-way ANOVA with Fisher’s LSD *post hoc* test for (*B*–*E*); ∗∗∗∗*p* < 0.0001, ∗∗∗*p* < 0.001, *ns*, nonsignificant. Cre, cAMP response element; FACS, fluorescence-activated cell sorting; LSD, least significant difference; MFI, mean fluorescence intensity; TSQ, 6-methoxy-8-p-toluenesulfonamido-quinoline.

Next, we assessed the specificity of βRepZnC-delivery to β-cells within primary islets, which contain multiple cell types. We hypothesized that βRepZnC accumulation, preferentially occurred in β-cells compared to other islet cells. To conduct this experiment, we isolated islets from Cre-reporter mTmG mice (*Ins2-Cre ROSA*^*mT/mG*^), which enabled independent examination of TSQ fluorescence and drug accumulation in β-cells (mEGFP-positive) and non-β-cells (mTomato-positive) by FACS sorting and analysis (Figs. 3*A* and S2). TSQ fluorescence was detected in β-cells and, to a lesser extent, non-β-cells (Figs. 3*B* and S3) (43); accordingly, GR-4661 treatment significantly and preferentially increased TSQ fluorescence in β-cells. Notably, GR-46611 did not significantly affect TSQ fluorescence in non-β-cells, demonstrating its selective action on β-cells.

Based on β-cell-selective amplification of zinc accumulation in response to GR-46611, we next tested the effects on βRepZnC accumulation. Accordingly, isolated mTmG islets were pretreated with vehicle or GR-46611, loaded with βRep compounds, and then sorted to measure [βRepZnC] per 1000 β-cells or non-β-cells. GR-46611 treatment did not impact islet cell composition, with β-cells comprising approximately 66% of the islet cells (Fig. 3*C*). Indeed, the selective effect of GR-46611 on β-cells was confirmed by the significant increase in βRepZnC accumulation in GR-46611-treated β-cells compared to vehicle-treated β-cells, with no effect observed in non-β-cells (Fig. 3*D*). The concentration of βRepZnC in sorted β-cells was 160-fold higher than that of the parent molecule GNF-4877, and this magnitude increased to ∼4000-fold with GR-46611 treatment (Fig. 3*D*).

This experiment revealed that (1) only zinc-chelating βRepZnC was efficiently retained by islet cells over the duration of experimentation (from islet dispersion through FACS), (2) the pronounced islet accumulation of βRepZnC was primarily *via* β-cell accumulation and (3) GR-46611 treatment selectively increased βRepZnC accumulation in β-cells. Accordingly, when assessing the ratio of drug accumulation in β-cells to non-β-cells, βRepZnC exhibited 3-fold β-cell selectivity, whereas the nonchelating molecules exhibited no preference (Fig. 3*E*). Furthermore, GR-46611 treatment enhanced β-cell βRepZnC accumulation without affecting non-β-cell accumulation (yielding a ∼5-fold β-cell selectivity) and had no effect on nonchelating compound accumulation (Fig. 3*E*). Taken together, these results demonstrated that islet accumulation of βRepZnC was zinc-dependent and β-cell-selective, and that these properties were enhanced by pharmacologically increasing β-cell zinc levels with GR-46611 treatment.

### GR-46611 increases ZnT8-mediated granular zinc *via* V-ATPase-driven granular acidification

**Figure 4:**
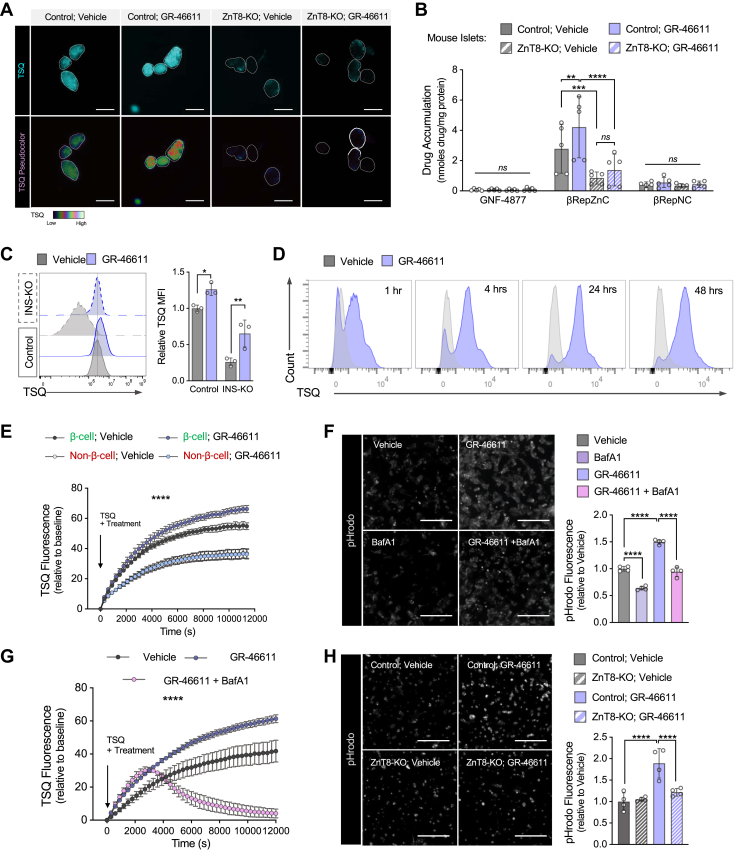
**GR-46611 enhances ZnT8-dependent zinc import into insulin granules through stimulation of V-ATPase–driven acidification in β-cells.***A*, live-fluorescence imaging of TSQ-stained *ZnT8*^*flox/flox*^ (Control) and *Ins2-Cre, ZnT8*^*flox/flox*^ (ZnT8-KO) mouse islets following vehicle or 10 μM GR-46611 treatment for 24 h. *B*, βRep drug accumulation measurements in Control and ZnT8-KO mouse islets pretreated with vehicle or GR-46611 (*n* = 5 mice/genotype). Data represent mean concentration normalized to protein concentration. *C*, FACS histograms of TSQ analysis in Control and INS-KO R7T1 β-cell treated with vehicle or GR-46611 treatment for 48 h. Data represent mean TSQ MFI relative to control vehicle (*n* = 3). *D*, FACS histograms of TSQ analysis in mouse islets treated with vehicle or GR-46611 treatment for 1, 2, 24, or 48 h. Data represent mean TSQ MFI relative to control vehicle (*n* = 3). *E*, time-lapse of TSQ analysis in non-β-cells and β-cells of dispersed mTmG mouse islet cultures following vehicle or GR-46611 treatment (n = 4 mice). Data represent mean TSQ fluorescence relative to baseline ± SEM. *Arrow* indicates time 0 at which TSQ and treatments were added. *F*, representative images of pHrodo fluorescence (*gray* scale) in dispersed mouse islet cultures treated with vehicle, GR-46611 and/or 100 nM bafilomycin A1 (BafA1) (*n* = 4 mice). Data represent pHrodo fluorescence intensity relative to vehicle. *G*, time-lapse of TSQ analysis in dispersed mTmG mouse islet cultures following vehicle, GR-46611 or BafA1 treatment (*n* = 3 mice). Data represent mean TSQ fluorescence relative to baseline ± SEM. *Arrow* indicates time 0 at which TSQ and treatments were added. *H*, representative images of pHrodo fluorescence (*gray scale*) in dispersed Control or ZnT8-KO mouse islet cultures treated with vehicle or GR-46611. Data represents pHrodo fluorescence intensity relative to control vehicle (*n* = 4 mice/genotype). The scale bars represent 100 μm. Data represent mean ± SD unless noted otherwise. Two-way ANOVA with Fisher’s LSD *post hoc* test for (*B*, *C*, *F*, and *H*); ordinary two-way ANOVA for (*E* and *G*). ∗∗∗∗*p* < 0.0001, ∗∗∗*p* < 0.001, ∗∗*p* < 0.01, ∗*p* < 0.05, *ns*, non-significant. Cre, cAMP response element; FACS, fluorescence-activated cell sorting; LSD, least significant difference; MFI, mean fluorescence intensity; TSQ, 6-methoxy-8-p-toluenesulfonamido-quinoline.

To determine whether GR-46611–induced zinc accumulation in β-cells requires ZnT8, we first examined TSQ fluorescence in ZnT8-deficient R7T1 β-cells and found that GR-46611 failed to increase TSQ fluorescence (Fig. S4), indicating that ZnT8 is required for zinc accumulation. We observed a similar requirement in β-cell–specific ZnT8-knockout islets (*Ins2-Cre, ZnT8*^*flox/flox*^), which showed reduced basal TSQ fluorescence and no response to GR-46611 treatment (Fig. 4*A*). Concordantly, ZnT8-knockout islets demonstrated substantially reduced βRepZnC accumulation, compared to control islets, and treatment with GR-46611, which increased βRepZnC accumulation in control islets, failed to increase βRepZnC accumulation in ZnT8-knockout islets (Fig. 4*B*). These data indicated that islet βRepZnC accumulation, and GR-46611-dependent augmentation of islet βRepZnC accumulation, were dependent upon β-cell expression of ZnT8. Consistent with this assertion, islet accumulation of the nonchelating drugs was unaffected by disruption of ZnT8 expression, or GR-46611 exposure (Fig. 4*B*). These findings demonstrate that GR-46611-mediated enhancement of zinc content and βRepZnC delivery is dependent on ZnT8 expression.

Given the zinc-complexing properties of insulin within β-cell granules (36), we hypothesized that insulin expression might be required for basal, and/or GR-46611-induced β-cell zinc accumulation. To test whether ZnT8-mediated zinc accumulation is influenced by insulin content, we assessed zinc levels in insulin-deficient R7T1 β-cells (INS-KO) by measuring TSQ fluorescence by FACS. Consistent with insulin’s known zinc-sequestering activity, INS-KO cells exhibited ∼4-fold lower basal TSQ fluorescence compared to control cells (Fig. 4*C*). However, GR-46611 increased TSQ fluorescence to a similar extent in both control and INS-KO β-cells (Fig. 4*C*), indicating that GR-46611-induced zinc accumulation is independent of insulin expression.

To further assess zinc dynamics, we monitored TSQ fluorescence over time in intact islets by FACS (Fig. 4*D*). GR-46611 treatment induced a time-dependent increase in zinc fluorescence, with a significant rise within 1 h and a peak at 24 h (Fig. 4*D*), indicating a rapid net increase in intracellular zinc content. Although these findings are consistent with enhanced zinc flux, we cannot exclude the possibility that reduced zinc efflux also contributes to the observed accumulation. Notably, the rapid onset of zinc augmentation within 1 h argues against slower adaptive processes such as increased granule biogenesis or reduced granule exocytosis as primary drivers of this response. To validate the effects on β-cell zinc flux, we performed time-lapse live-cell imaging of TSQ labeling using dispersed mTmG islet cultures treated with TSQ and vehicle or GR-46611 (Fig. 4*E* and S5). As expected, β-cells exhibited approximately double the TSQ fluorescence intensity of non-β-cells (Figs. 4*E* and S5; Video S1). In non-β-cells, no significant difference in TSQ fluorescence was observed between vehicle- and GR-46611-treated conditions (Figs. 4*E* and S5; Video S1, S2). Given GR-46611 dependence on ZnT8 granular expression, these data raised the possibility that GR-46611 induced a higher steady-state granular zinc concentration by increasing the driving force for zinc into the granule lumen.

Given ZnT8’s proposed role as a proton-coupled zinc transporter (21, 31, 32, 44, 45), we examined whether GR-46611 augments zinc flux through vesicle acidification. Zinc flux into β-cell granules is tightly linked to acidification of secretory granules, which is primarily established by vacuolar ATPase (V-ATPase) activity, an ATP-dependent proton pump (46). To determine if GR-46611-induced increase in granular zinc levels might be reflected by V-ATPase activity, we monitored intracellular acidification in dispersed mouse islet cells using the pH-sensitive dye, pHrodo. Indeed, GR-46611 treatment significantly increased pHrodo fluorescence by live-cell imaging (Fig. 4*F*) and by FACS (Fig. S6), indicating enhanced acidification. We note that GR-46611-treatment had no significant effect on side scatter, an indirect measurement of insulin granule content (Fig. S6*C*). Moreover, treatment with bafilomycin A1, a V-ATPase inhibitor, reduced both basal and GR-46611-induced acidification (Fig. 4*F*). Consistently, bafilomycin A1 treatment for 4 h significantly depleted TSQ fluorescence in GR-46611-treated primary β-cells (Fig. 4*G*; Video S3–S5), demonstrating that V-ATPase activity was critical for maintaining GR-46611-dependent zinc flux. As an additional indication of V-ATPase engagement, immunoblot analysis of V-ATPase subunits showed a selective increase in the V0 domain (which mediates proton translocation), but not the V1 domain, following GR-46611 treatment (Fig. S7), supporting a role for enhanced proton pumping and thereby, vesicle acidification in driving zinc uptake.

To test whether this acidification response is functionally coupled to ZnT8-mediated zinc transport, we examined pHrodo fluorescence in ZnT8-KO islets. Basal acidification was intact, but GR-46611 failed to increase pHrodo signal in ZnT8-KO cells (Fig. 4*H*), indicating that ZnT8 is required for the acidification response. Interestingly, V-ATPase V0 expression was elevated at baseline in ZnT8-KO islets and was not further increased by GR-46611 (Fig. S7), perhaps suggesting a potential feedback relationship between ZnT8 and V-ATPase activity. Together, these findings revealed a tightly coupled mechanism in which GR-46611 promoted ZnT8-dependent zinc flux into insulin granules by enhancing V-ATPase–driven vesicle acidification.

### Inhibiting cAMP-PKA signaling enhances granule acidification, zinc flux, and βRepZnC accumulation

Although the genetic and signaling mechanisms regulating ZnT8 activity remain largely unknown, pathways that induce luminal acidification—and, in the context of β-cells, might promote granular zinc accumulation—have been studied in other model systems (47). Among identified pathways, inhibition of cAMP signaling is proposed to induce vesicular acidification (48). In addition, GR-46611 has been characterized as a relatively selective inhibitory G-protein coupled receptor (G_i/o_) serotonin (5-hydroxytryptamine; 5-HT) receptor 1D (5-HTR1D) agonist (49). Accordingly, we hypothesized that inhibition of cAMP signaling would increase β-cell granule acidification and zinc accumulation. To test this notion, we evaluated the effects of GR-46611 on cAMP pathway activity.

**Figure 5:**
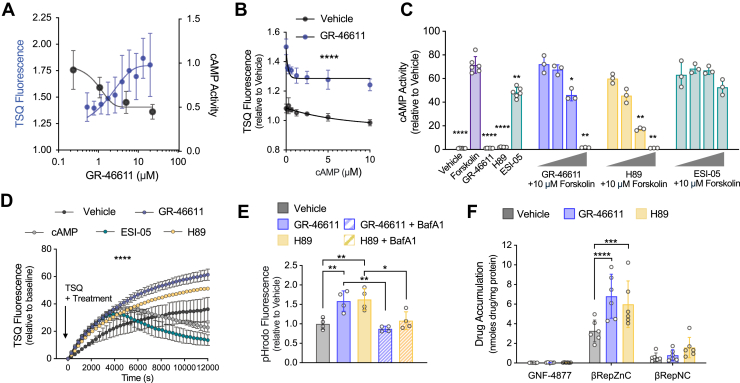
**Enhanced zinc flux, granule acidification, and βRepZnC accumulation are mediated by cAMP-PKA inhibition.***A*, dose curve of TSQ fluorescence (*blue*) and luciferase-based cAMP activity (*black*) in response to GR-46611 treatment for 48 h in growth-arrested R7T1 β-cells. *B*, dose-dependent effects of cAMP treatment for 24 h on TSQ fluorescence in growth-arrested R7T1 β-cells. *C*, luciferase-based cAMP activity in response to increasing concentrations of compounds (1, 5, 10, and 25 μM). Cells were either treated with compound alone or pretreated for 4 h prior to stimulation with 1 μM forskolin. *D*, time-lapse of TSQ analysis in dispersed mTmG mouse islet cultures following vehicle, 10 μM GR-46611, 10 μM cAMP, 10 μM ESI-05 or 10 μM H89 treatment (*n* = 3 mice). Data represent mean TSQ fluorescence relative to baseline ± SEM. *Arrow* indicates time 0 at which TSQ and treatments were added. *E*, quantification pHrodo fluorescence intensity in dispersed mouse islet cultures treated with compounds ± BafA1 for 4 h (*n* = 4 mice). *F*, βRep drug accumulation measurements in mouse islets pretreated with vehicle, GR-46611 or H89 (*n* = 6 mice). Data represent mean concentration normalized to protein concentration. The scale bars represent 100 μm. Data represent mean ± SD unless noted otherwise. Uncorrected Fisher’s LSD compared to forskolin for *C*; Ordinary two-way ANOVA for D; two-way ANOVA with Fisher’s LSD *post hoc* test for (*E* and *F*); ∗∗∗∗*p* < 0.0001, ∗∗∗*p* < 0.001, ∗∗*p* < 0.01, ∗*p* < 0.05, *ns*, nonsignificant. LSD, least significant difference; TSQ, 6-methoxy-8-p-toluenesulfonamido-quinoline.

In β-cells, cAMP is a critical mediator of insulin release, modulating pathways that are both glucose-dependent and glucose-independent (50, 51, 52). Interestingly, GR-46611 pretreatment did not impair glucose-stimulated insulin secretion in isolated mouse islets when applied prior to the assay and removed before stimulation (Fig. S8), suggesting no major effect on insulin content, granule availability or secretory machinery. Next, we investigated the impact of GR-46611 on cAMP signaling and its relationship with zinc accumulation. Using a luciferase-based cAMP reporter assay in R7T1 β-cells, we found that GR-46611 reduced transcriptional activity of the cAMP response element (CRE) in a dose-dependent manner that anticorrelated with an increase in TSQ fluorescence (Fig. 5*A*). Conversely, direct treatment with cAMP dose-dependently decreased TSQ fluorescence (Fig. 5*B*), indicating an inverse relationship between cAMP signaling and β-cell zinc content.

Having implicated the cAMP pathway in regulating β-cell zinc accumulation, we next interrogated the two primary downstream effectors of cAMP: protein kinase A (PKA) and Epac2 (51). First, to confirm pathway selectivity, we assessed the effects of H89 and ESI-05—inhibitors of PKA and Epac2, respectively—on CRE-luciferase activity. In this assay, compounds were applied prior to stimulation with forskolin—a direct activator of adenylyl cyclase—to evaluate their ability to suppress cAMP–PKA signaling. Forskolin robustly induced CRE activity, which was significantly suppressed by GR-46611 and H89 in a dose-dependent manner (Fig. 5*C*). In contrast, ESI-05 had no effect on CRE activity, consistent with its specificity for the non-PKA arm of cAMP signaling.

Next, we assessed zinc dynamics by treating dispersed mTmG islets with selective inhibitors and performing time-lapse imaging of TSQ fluorescence. (Figs. 5*D* and S9*A*; Video S6-10). As hypothesized, cAMP treatment reduced steady state zinc levels (Video S8). Interestingly, ESI-05 treatment also reduced steady-state zinc content (Video S9), suggesting a potential role for Epac2 in maintaining granular zinc content, or possibly a compensatory increase in cytosolic cAMP. In contrast, PKA inhibition with H89 significantly enhanced zinc accumulation (Video S10), indicating that PKA activity negatively regulates β-cell zinc levels. This finding was further supported by similar results with the PKA-selective inhibitor Rp-8-Br-cAMPS, which also suppressed cAMP–PKA signaling and increased zinc levels (Fig. S9, *B* and *C*).

The mirrored effects of GR-46611 and H89 on β-cell zinc accumulation suggested a shared mechanism of action through PKA pathway inhibition. To test this further, we assessed their effects on vesicle acidification and found that both GR-46611 and H89 significantly increased acidification in primary islet cultures (Fig. 5*E*). Moreover, V-ATPase inhibition with bafilomycin A1 blocked pHrodo induction in both GR-46611- and H89-treated β-cells (Fig. 5*E*), confirming the role of V-ATPase in mediating this response. As additional evidence of V-ATPase activation, both GR-46611 and H89 increased expression of the V0 subunit, which mediates proton translocation (Fig. S7). Taken together, these results indicated that the mechanism of GR-4661-induced β-cell granule acidification and zinc accumulation was *via* cAMP-PKA pathway inhibition.

To further interrogate this conclusion, we evaluated whether cAMP-PKA pathway inhibition with H89 promoted βRepZnC accumulation in mouse islets, as observed with GR-46611 treatment. Indeed, H89 and GR-46611 treatment similarly amplified islet βRepZnC accumulation, resulting in a 2-fold increase relative to the control (Fig. 5*F*). The accumulation of nonchelating compounds was unaffected by GR-46611 or H89 treatment (Fig. 5*F*). Collectively, these results indicated that cAMP-PKA pathway inhibition promoted β-cell zinc accumulation *via* V-ATPase-driven granule acidification.

### β-cell zinc selectively and durably potentiates βRepZnC-induced β-cell replication

**Figure 6:**
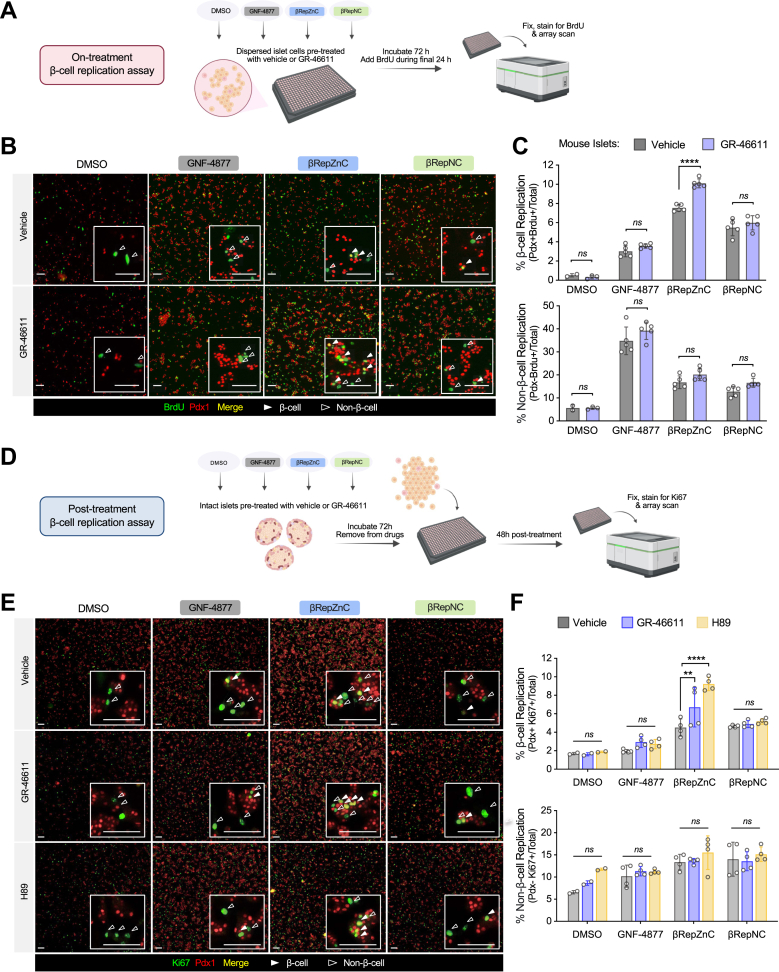
**βRepZnC replication efficiency increases with zinc elevation and persists after drug removal.***A*, schematic representation of “on-treatment” β-cell replication assay. Dispersed mouse islets were pretreated with vehicle or GR-46611 followed by addition of 1.5 μM βRep drugs for 72 h and immediately fixed for immunostaining. *B*, representative images of dispersed mouse islet cultures immunostained with Pdx1 (*red*) and BrdU (*green*). *White arrows* indicate replicating β-cells (Pdx1-positive), *hollow arrows* indicate replicating non-β-cells (Pdx1-negative). *C*, β-cell replication and non-β-cell replication rates in mouse islets (*n* = 5 mice). *D*, schematic representation of “post treatment” β-cell replication assay. Intact islet cultures were pretreated with vehicle or GR-46611 followed by addition of 1.5 μM βRep drugs for 72 h. Treated islets were then dispersed and plated for a 48-h washout period prior to fixation and analysis. *E*, representative images of dispersed mouse islet cultures immunostained with Pdx1 (*red*) and Ki-67 (*green*). *White arrows* indicate replicating β-cells (Pdx1-positive), *hollow arrows* indicate replicating non-β-cells (Pdx1-negative). *F*, β-cell replication and non-β-cell replication rates in mouse islets (*n* = 4 mice). The scale bars represent 100 μm. Data represent mean ± SD unless noted otherwise. Two-way ANOVA with Fisher’s LSD *post hoc* test for (*C* and *F*); ∗∗∗∗*p* < 0.0001. LSD, least significant difference

Finally, the substantial increase in βRepZnC accumulation with zinc-elevating treatment suggested that pharmacologic zinc augmentation might selectively enhance the replication-promoting activity of βRepZnC. To test this possibility, we measured primary β-cell and non-β-cell replication using an ”on-treatment” assay (Fig. 6*A*), capturing the replication effects of βRep compounds after a 72-h treatment period in dispersed mouse islet cells that were pretreated with vehicle or GR-46611. In this method, BrdU—a thymidine analog incorporated during DNA synthesis—was added during the final 24 h of βRep compound treatment. GR-46611 alone did not increase β-cell or non-β-cell replication in vehicle-treated samples, nor did it enhance replication induced by GNF-4877 or βRepNC; however, GR-46611 significantly enhanced β-cell replication induced by βRepZnC (Fig. 6, *B* and *C*). Moreover, GR-46611’s effect remained specific to β-cells, with no changes in replication observed in non-β-cell populations. A similar zinc-dependent enhancement of βRepZnC-induced β-cell replication was also observed in human islet cells (Fig. S10).

Because βRepZnC accumulation was significantly retained in sorted β-cells (Fig. 3*F*), we next asked whether zinc elevation exerted an enduring effect on β-cell replication, even after withdrawal of βRepZnC treatment (Fig. 6*D*). In this “post-treatment” assay, intact mouse islets were treated with βRep compounds for 72 h, followed by a 48-h washout period. β-cell replication was then assessed using Ki67, a transiently expressed marker of actively cycling cells, enabling detection of sustained proreplicative effects of βRepZnC, post treatment.

Both GR-46611 and H89 selectively enhanced βRepZnC-induced β-cell replication, increasing rates from 4.5% (vehicle) to 6.7% and 9.2%, respectively, with no effects observed in cocultured non-β-cells (Fig. 6, *E* and *F*). Strikingly, although GNF-4877 induced β-cell replication in the on-treatment assay (concurrent drug exposure), its effect was abolished in the post treatment assay, where β-cell replication returned to baseline levels (2.0% with vehicle, 2.9% with GR-46611), comparable to dimethyl sulfoxide (DMSO) controls (1.7%). In contrast, βRepZnC exhibited sustained β-cell replication induction post treatment, reinforcing the selective and enduring activity of this zinc-dependent compound. Using this post treatment assay, we demonstrated that pharmacologic elevation of zinc—achieved by inhibiting the cAMP-PKA pathway—enhanced βRepZnC delivery and selectively improved β-cell replication efficiency.

## Discussion

Previously, we reported a unique zinc-based platform technology for β-cell-targeted drug delivery (18). The approach integrated a zinc-chelating moiety and a β-cell replication compound, generating βRepZnC, which preferentially accumulates and functions within β-cells. The strategy was purported to depend upon the uniquely high zinc concentration of β-cells; however, this conclusion fully rested on the ability of a zinc chelator, TPEN, to abrogate islet βRepZnC accumulation. In this study, we provide robust orthogonal experimental evidence in support of a zinc-based β-cell-targeted drug delivery platform. First, we leveraged genetically engineered islets (β-cell-specific ZnT8-KO) to firmly demonstrate islet βRepZnC accumulation is dependent upon the uniquely high granular zinc content of β-cells. Second, we discovered pharmacologic methods to enhance β-cell zinc content (GR-46611 and H89) and, using these compounds, showed that pharmacologically increasing β-cell zinc content also augmented islet βRepZnC accumulation. Third, we developed enhanced assays to demonstrate that βRepZnC preferentially accumulated within β-cells, as opposed to non-β-cells of the same islet. Fourth, we demonstrated that the β-cell-selectivity of replication induction was enhanced by combining βRepZnC with pharmacologic inducers of β-cell zinc content, resulting in an enduring replication effect that persists beyond treatment withdrawal. Finally, we demonstrate that this βRepZnC retention is achieved by enhancing β-cell zinc *via* a previously unrecognized mechanism, whereby inhibition of cAMP–PKA signaling promotes ZnT8-mediated zinc transport into secretory granules, functionally coupled to V-ATPase–driven vesicle acidification. Together, these findings advance a two-pronged therapeutic strategy: (1) pharmacologic elevation of β-cell zinc through targeted modulation of vesicle zinc flux, and (2) leveraging of the zinc-enriched environment for selective delivery, retention and activity of β-cell–targeted regeneration therapeutics.

A primary discovery of this work is the identification of GR-46611 as a potent inducer of β-cell zinc accumulation in both mouse and human islets. Through mechanistic studies, we also identified H89 (and other PKA inhibitors) as a pharmacological stimulator of β-cell zinc content. These compounds enhanced granular zinc, a process dependent on granular acidification and ZnT8 expression. GR-46611 is considered a relatively selective agonist for the inhibitory (G_i/o_-coupled) serotonin (5-hydroxytryptamine; 5-HT) receptor 1D (5-HTR_1D_) that is expressed in α-, β-, and δ-cells of human islets (49, 53, 54). Yet, we found that the 5-HT_1A,B,D_ receptor agonist sumatriptan and the 5-HT_1D_-selective agonist PNU 142633 (5-HT_1D_ >>100–1000 fold 5-HT_1A,B_) (55) had no effect on zinc content (data not shown). Thus, the precise molecular target(s) for GR-46611-induced β-cell zinc accumulation remain to be better defined. Interestingly, GR-46611 induction of β-cell zinc accumulation was independent of insulin expression, although insulin-deficient cells demonstrated reduced baseline levels of zinc. Diabetic patients have reduced islet insulin content, potentially underlying the depletion of islet zinc observed in diabetes (56, 57, 58). Thus, in the context of diabetes, zinc-dependent drug targeting to β-cells could be improved by pharmacologically increasing β-cell zinc with a compound like GR-46611 and/or through insulin therapy to promote β-cell rest, boost insulin content and thus, zinc levels.

Beyond GR-46611, our findings reveal a broader role for cAMP–PKA signaling in regulating intracellular zinc content in β-cells—a novel mechanistic insight into zinc homeostasis. Although zinc is known to be highly abundant in β-cells and critical for insulin crystallization and secretion (21, 25, 26), the upstream signaling pathways that regulate zinc accumulation remain poorly defined. Previous studies have implicated factors such as glucose metabolism, reactive oxygen species (ROS), and transcriptional control of ZnT8 (*SLC30A8*) as modulators of β-cell zinc content (59, 60). However, direct links between intracellular signaling cascades and dynamic zinc flux have not been established. Our study identifies the cAMP–PKA pathway as a key upstream regulator of β-cell zinc dynamics. Pharmacological modulation of this pathway revealed that its inhibition enhances β-cell zinc accumulation, whereas activation suppresses it. This bidirectional effect was consistently observed across diverse agents, including H89, and Rp-8-Br-cAMPS (inhibitors) and forskolin and exendin-4 (activators). These findings not only advance our understanding of β-cell zinc biology but also uncover a tractable signaling axis that can be modulated to enhance zinc-dependent drug delivery. Given the central role of cAMP–PKA signaling in β-cell function and glucose homeostasis (50, 51, 52), the ability to fine-tune zinc levels through this pathway opens new avenues for targeted therapeutic strategies.

Another key mechanistic finding of this work is the identification of a regulatory mechanism for ZnT8-mediated granular zinc transport in β-cells. While ZnT8, is proposed to function as a zinc-2H^+^ antiporter in β-cell insulin granules, this conclusion is primarily derived from structural inference (31, 32). Our data provide functional validation by demonstrating that β-cell granular zinc content is tightly coupled to vesicle acidification, establishing a physiologic link between ZnT8 activity and V-ATPase function. This connection reinforces previous observations that β-cell secretory granules are acidified by V-ATPase, lowering their pH to approximately 5.5 (21, 44) and that zinc transport is coupled to V-ATPase activity (61, 62). Notably, we show that GR-46611-induced vesicular acidification is accompanied by a selective increase in the V0 subunit of V-ATPase—the proton-translocating domain—while V1 subunit levels remain unchanged. We propose that V0 subunit expression (or stabilization) is linked to ZnT8 expression, as evidenced by the upregulation of V0 subunit (but not V1) in ZnT8-KO islets. Interestingly, V-ATPase function requires both the V0 and V1 subunits; hence, it is unclear how selective induction of the V0 subunit might increase granule acidification. One possibility is that these subunits are not stoichiometrically expressed or assembled at baseline, *i.e.* the V1 subunit may be present in excess, allowing V0 expression or localization to serve as a control point for acidification. Although this supposition remains theoretical, our findings demonstrate a previously unrecognized regulatory connection between ZnT8, V-ATPase V0 and vesicular acidification in β-cells.

Targeted β-cell regeneration therapies require both selective and sustained biological activity, yet current approaches fall short on these fronts. GNF-4877, a potent DYRK1A inhibitor that induces β-cell replication, has substantial off-target replication effects on nonislet cells that limit its potential utility (3). Moreover, to achieve *in vivo* efficacy, nontargeted replication drugs like GNF-4877 must be maintained at therapeutic (bioactive) concentrations throughout the body, which exacerbates undesirable off-target activity—highlighting the need for strategies that maximize efficacy while minimizing off-target drug exposure (10, 18, 63). To address these limitations, we developed a zinc-targeted delivery system using βRepZnC. Strikingly, in addition to preferential accumulation of βRepZnC in β-cells, we found that its replication-promoting activity—unlike non-zinc-binding replication drugs—persisted for at least 48 h after drug removal and was β-cell selective. In principle, this unique pharmacokinetic behavior further enhances the *in vivo* β-cell selectivity of βRepZnC: transient systemic exposure limits off-target bioactivity but enables βRepZnC bioaccumulation within islets, where it is expected to exert sustained, localized, and targeted replication-promoting activity. Thus, we propose that β-cell bioaccumulation of βRepZnC might effectively transforms β-cells into a reservoir of βRepZnC, providing localized and sustained, islet-targeted drug delivery and bioactivity. As a result, an intermittent and transient βRepZnC dosing schedule may be leveraged to minimize off-target replication-promoting activity of DYRK1A inhibitors.

Interestingly, recent single-cell RNA sequencing analysis of harmine-induced human β-cell replication raised the possibility that cycling α-cells, which purportedly transdifferentiate into β-cells, were the primary *in vivo* human islet therapeutic target (64, 65). These findings are consistent with prior reports suggesting basal human α-cell replication is ∼5-fold that of the very low basal human β-cells replication rate (∼0.16%) (66). Accordingly, for the purpose of β-cell regeneration (but not necessarily for the purposes of β-cell preservation or other indications), stringent β-cell-specific targeting could be counterproductive. Thus, it is notable that βRepZnC preferentially accumulated, but to a lesser extent, in islet non-β-cells relative to untargeted replication compounds, reflecting the appreciable zinc content present in other islet cell types such as α- and δ-cells (43). Hence, future work might assess whether βRepZnC are effectively stored in β-cells and, less efficiently retained in ZnT8-expressing α-cells, potentially providing biologically relevant levels of drug exposure to both β-cells and adjacent islet endocrine cells (particularly in an *in vivo* context). To advance the field, rigorous testing of selective drug accumulation and bioactivity *in vivo* will be essential for optimizing zinc-dependent therapeutic delivery and induction of β-cell regeneration.

In addition to the noteworthy advances described above, a few limitations of our study should be recognized. First, while our results provide a strong foundation for the development of β-cell-targeted regenerative therapies, a limitation of this study is the lack of *in vivo* testing to evaluate the systemic administration of these compounds and their effects on β-cell replication. Other cell types with higher-than-average zinc content, such as α-cells, Paneth cells, and certain neurons, must be considered as potential targets (67, 68, 69, 70). However, since β-cells contain significantly higher zinc levels (>8-fold higher) compared to these cell types (71), we expect zinc-chelating drugs like βRepZnC to exhibit greater potency and efficacy toward β-cells and potentially other islet cells. Moreover, GR-46611's effect on zinc is dependent on the presence of ZnT8, a transporter expressed in a limited number of cell types, predominantly in β-cells (67, 72). Second, an important question remains regarding how βRepZnC, once compartmentalized in insulin granules, is able to reach and inhibit DYRK1a, which functions in cellular compartments outside the granules. In addition, future studies exploring how glucose-stimulated insulin secretion influences βRepZnC accumulation and bioactivity are important for understanding the cellular uptake and release of βRepZnC. Hence, the pharmacokinetics and precise mechanisms governing βRepZnC’s intracellular uptake, retention, and release require further investigation. Third, while we demonstrate enhanced β-cell replication across multiple human islet donors, mechanistic studies—including zinc content or drug accumulation assays— independent validation by other laboratories using additional human donor samples in *in vitro* and *in vivo* models will be important to further assess the translational and therapeutic potential of our technologies.

In summary, the current work powerfully advances the concept of zinc-powered β-cell-targeted drug delivery, provides new molecular insights into pathways regulating β-cell zinc content, and highlights the potential to pharmacologically augment these pathways to achieve β-cell-targeted delivery of βRepZnCs to reverse β-cell loss in diabetes.

## Experimental Procedures

### R7T1 β-cells

R7T1 is a reversibly immortalized mouse β-cell line that expresses SV40 T antigen under a tetracycline inducible promoter (39). R7T1 β-cells (authenticated; *mycoplasma* tested) were expanded/cultured in Dulbecco's modified Eagle's medium (DMEM)/high glucose media (Cytiva) containing 4.0 mM L-glutamine, 1 mM sodium pyruvate, 10% fetal bovine serum (FBS), 1 IU/ml penicillin, 1 mg/ml streptomycin, and 10 μg/ml doxycycline. DMEM has been reported to contain approximately 1.5 nM zinc (https://www.sigmaaldrich.com/US/en/technical-documents/technical-article/cell-culture-and-cell-culture-analysis/mammalian-cell-culture/zinc) and 10% FBS contributes an estimated zinc concentration of 0.25 nM as reported previously (73). R7T1 cells were growth arrested in doxycycline-free R7T1 medium for 5 days prior to all *in vitro* experiments. Cells were maintained in a 37 °C humidified incubator with 5% CO_2_.

### Human islets

Human islets for research were provided by the Alberta Diabetes Institute IsletCore at the University of Alberta in Edmonton (http://www.bcell.org/adi-isletcore.html) with the assistance of the Human Organ Procurement and Exchange (HOPE) program, Trillium Gift of Life Network (TGLN), and other Canadian organ procurement organizations. Islets were cultured overnight in DMEM/low glucose (Cytiva) with 4.0 mM L-glutamine, 1 mM sodium pyruvate, 10% FBS, 1 IU/ml penicillin, 1 mg/ml streptomycin (islet culture medium) before experiments.

### Rodent islets

Primary islets were isolated from C57BL/6J mice (Jax #00064) and Ins2-Cre mice (Jax #003573) crossed with either fluorescent Cre-reporter mice ROSA^mT/mG^ (Jax # 007676) or ZnT8 flox/flox mice (74). Sex was not considered as a biological variable in this study. Our study analyzed primary islets from both male and female mice. Rodents were housed in ventilated cages with access to water and normal chow ad libitum. Islet isolation was performed by pancreatic perifusion of Cizyme (VitaCyte) and purification with Histopaque (Sigma-Aldrich) density gradient centrifugation as previously described (9, 75). Isolated islets were cultured overnight in islet medium before experiments. For each treatment condition, ∼50 islets from the same mouse were used to control for intermouse variability. All intact islet assays were performed in nontreated 12-well plates (CELLTREAT Scientific Products).

### Zinc-based chemical screen

Growth-arrested R7T1:H2B-GFP cells were plated in 384-well plates at 17,000 cells/well and treated in duplicates with the LOPAC 1280 compound library (Sigma-Aldrich LO1280) for 48 h at 10 μM and 1 μM according to manufacturer’s recommended concentration. Each plate included cells treated with 0.1% DMSO as vehicle control to avoid plate variability. For the primary screen, each well was loaded with a membrane-permeable fluorescent zinc probe, TSQ (λmax = ∼470 nm; final concentration 100 μM) at 37 °C for 30 min prior to automated imaging on the ArrayScan VTI HCS (Thermo Fisher Scientific). Cells were identified based on the nuclear GFP signal. Image analysis was performed using the Cellomics software. TSQ fluorescence intensity was quantified in the cytoplasmic ring of each cell and the intensity relative to vehicle treatment (baseline control) was used as the primary outcome metric. Compounds were designated as hits if mean TSQ fluorescence was greater than 2.5 standard deviations from that of vehicle control on each plate (calculated z-score > 2.5). Initial hits were validated by retesting in replicates of seven and are summarized in Table S1. Further information on key reagents used in this study are listed in Table S2.

### Flow cytometry

Growth-arrested R7T1 cells were plated in 6-well format, treated with compounds or 0.1% DMSO (vehicle control) for 24 to 48 h, trypsinized and resuspended in FACS buffer (5% FBS in PBS) containing specific fluorophores for live-cell staining at 37  °C for 30 min: TSQ (100 μM; 360/490 nm; MedChemExpress). Prior to analysis, cell viability dye, propidium iodide was loaded for 15 min on ice. Stained cells were analyzed on the BD LSRII.UV flow cytometer (50–100 K events) and data were processed in FlowJo v10 software. Data were represented as average mean fluorescence intensity (MFI) of TSQ relative to vehicle control (Relative TSQ MFI). For primary β-cells, isolated islets (50 islets) from Ins2-cre; ROSA^mT/mG^ mice were treated with 10 μM GR-46611 or vehicle control, dispersed with Accutase (Innovative Cell Technologies), and then resuspended in FACS buffer containing TSQ for analysis. TSQ MFI was determined in mG-positive (β-cells) and mT-positive (non-β-cells) gated populations. Gating schemes are shown in Fig. S2.

### Live-cell TSQ imaging

Proliferating or growth-arrested R7T1 cells were plated in a black/clear bottom 96-well format, treated with compounds for 48 h, and then loaded with TSQ (100 μM diluted in PBS) at 37  °C for 30 min prior to automated imaging on the Operetta CLS (PerkinElmer). Cytoplasmic TSQ fluorescence intensity was quantified on the Harmony software. All representative images of treatment groups were captured using the “Overview-plate and well-realistic” for unbiased, identical image processing and comparison. For time-lapse analysis of live TSQ imaging, isolated mTmG islets were dispersed with 0.25% Trypsin/EDTA, plated at 25,000 cells/well into 384-well plates precoated with conditioned media from 804G rat bladder carcinoma cells and allowed to attach for 24 h. Dispersed islet cells were simultaneously loaded with treatment compounds and TSQ following immediate analysis on the Operetta CLS in the “Time Series” mode. TSQ fluorescence intensity fluorescence intensity was specifically quantified in β-cells (mEGFP-positive) and non-β-cells (mTomato-positive).

### Zinc content

Primary islets (∼100 islets per condition) were treated with 10 μM GR-46611 (Tocris) or vehicle control for 24 h, digested with concentrated nitric acid, heated at 100 °C for 30 s, and diluted to 2% nitric acid. Samples were analyzed by inductively coupled plasma-optical emission spectrometry on a iCAP 6300 Duo View Spectrometer (Thermo Fisher Scientific), and zinc concentrations were determined using zinc standards (ppm). An aliquot (10 μl) of digested sample was neutralized with equal volume of 2 N NaOH for protein quantification by bicinchoninic acid (BCA) protein assay. Data were represented as average zinc concentrations normalized to protein (Intracellular Zinc μmol/g protein).

### Islet drug accumulation

Primary islets (∼50 islets per condition) were pretreated with either 10 μM GR-46611, 10 μM H89 (MedChemExpress) or vehicle control in islet culture medium at 37 °C for 24 h. In the presence of pretreatment conditions, islets were loaded with either GNF-4877, βRepZnC, or βRepNC at a final concentration of 1.5 μM for 72 h. Drug delivery efficiency was tested by cotreatment of either βRepZnC or βRepNC with GNF-4877, providing an internal control for data normalization that accounted for potential confounding impacts of sample handling. Islets were washed in PBS two times, extracted with 50 μl lysis buffer (20 mM N-tris(hydroxymethyl)methyl-2-aminoethanesulfonic acid, 10 mM mannitol, and 10% Triton X-100) supplemented with protease and phosphatase inhibitor cocktail and frozen at −80  °C until analysis. Thawed samples were further extracted with 50 μl LCMS-grade acetonitrile, vortexed, and centrifuged at 10,000 rpm for 5 min. Supernatant was diluted 1:1 in 0.1% formic acid and analyzed by liquid chromatography–tandem mass spectrometry on a 6495C triple quadrupole mass spectrometer (Agilent), using a C18 column (Agilent) and methods as previously described (18, 42). Data were represented as average islet drug concentration normalized to protein (Drug accumulation nmoles/mg protein). To assess drug accumulation in β-cells and non-β-cells, isolated islets (50 islets) from Ins2-cre; ROSA^mT/mG^ mice were treated with 10 μM GR-46611 or vehicle control for 4 h, loaded with replication compounds for 72 h and then dispersed for sorting and collection of mG-positive (β-cells) and mT-positive (non-β-cells). Data were represented as average islet drug concentration per 1000 sorted cells. Reagents and compounds used in this study are listed in Table S2.

### Intracellular acidification

Primary islets were dispersed with 0.25% Trypsin/EDTA, plated at 25,000 cells/well into 384-well plates precoated with conditioned media from 804G rat bladder carcinoma cells and allowed to adhere for 24 h. Dispersed islets cells were treated with 10 μM GR-46611 or vehicle control in islet culture medium for 4 h and then loaded with pH-sensitive pHrodo AM Red for 30 min at 37  °C according to manufacturer’s protocol (Thermo Fisher Scientific; P35372). Cells were washed with PBS before imaging on Operetta CLS. pH-dependent changes in pHrodo fluorescence were confirmed in cells using pH Calibration Buffers (Fig. S6), and fluorescence intensity was quantified on the Harmony software.

### cAMP activity

Growth-arrested R7T1 or Hek293 cells were transfected with cAMP Response Element-firefly and cytomegalovirus-renilla as the coreporter vector (Promega) using Lipofectamine 2000 according to manufacturer’s protocol (Thermo Fisher Scientific). Post transfection, cells were replated in a 96-well plate and treated with 10 μM GR-46611 or vehicle control for 48 h. Cells were lysed with luciferase lysis buffer and luminescence was measured. Data were represented as average firefly over renilla luminescence (cAMP activity firefly/renilla).

### β-cell replication assay

β-cell replication was measured by two different complementary assays: on-treatment and post treatment. For on-treatment assay, intact primary islets were dispersed with 0.25% Trypsin/EDTA, plated at 25,000 cells/well into 384-well plates precoated with conditioned media from 804G rat bladder carcinoma cells and allowed to adhere for 24 h. Dispersed islet cells were pretreated with 10 μM GR-46611 or vehicle control in islet culture medium at 37  °C for 4 h. In the presence of pretreatment conditions, islets were loaded with either GNF-4877, βRepZnC, or βRepNC at a final concentration of 1.5 μM for 72 h, labeled with 5-Bromo-2′-deoxyuridine (BrdU; Thermo Fisher Scientific) at a final concentration of 1 μM during the last 24 h incubation period and then fixed for staining. For the post treatment assay, intact primary islets (∼150 islets per condition) were pretreated with either 10 μM GR-46611, 10 μM H89 or vehicle control in islet culture medium at 37  °C for 4 h. In the presence of pretreatment conditions, islets were loaded with either GNF-4877, βRepZnC, or βRepNC at a final concentration of 1.5 μM for 72 h. Islets were then removed from all treatment media, washed in PBS two times, dispersed, and plated. After 48 h to adhere (in the absence of drug), cells were fixed with 4% paraformaldehyde for 20 min at room temperature, antigen retrieved in freshly made sodium citrate/formamide buffer at 70  °C for 45 min and blocked in 5% donkey serum, 0.3% Triton-x 100/PBS for 1 h. Cells were stained with primary antibodies overnight- Pdx1 (Cell Signaling Technology; #56791:200), BrdU (Abcam; ab6326; 1:200), insulin (Cell signaling Technology; #3014; 1:500), and Ki-67 (BD Pharmingen; 550609; 1:200)-followed by 1 h incubation in secondary antibodies (Jackson ImmunoResearch), and nuclear staining (Hoechst 33342, 4 μM). Automated analysis was performed with Cellomics ArrayScan VTI HCS Reader. All antibodies used in this study are listed in Table S2 and control staining is provided in Fig. S11.

### Lentiviral transduction

Lentiviruses were generated in HEK293T cells with third generation packaging plasmids: pRSV-Rev, pMDLg/pRRE, and pMD2.G (Addgene). Expression vector virus was collected 48 h after transfection and filtered with a 0.45 μM pore filter prior to infecting R7T1 cells. For live-cell imaging on the high-content array scan, R7T1 β-cells were engineered to express nuclear GFP by lentiviral transduction using H2B-GFP plasmid. To generate INS-KO cells, Cas9-expressing R7T1 cells were first generated using lentiCas9-Blast. Single-guide RNA (sgRNA) sequences were cloned into pMCB320 vector. All single-guide RNA sequences are listed in Table S2.

### Immunoblotting

Primary islets (∼50 islets per condition) were lysed with RIPA buffer (50 mM Tris pH 7.4, 0.5% NP-40, 150 mM NaCl, and 0.1% SDS) containing protease and phosphatase inhibitor cocktail. Cell lysates (20 μg protein) were subjected to SDS-polyacrylamide gel electrophoresis using 4 to 15% gradient gel (Bio-Rad), transferred to nitrocellulose membrane by Trans-Blot Turbo Transfer and blocked in Odyssey TBS blocking buffer for 1 h. The following primary antibodies were incubated overnight: ATP6V0A1 (Sigma-Aldrich; SAB2108042; 1:1000), ATP6V1B2 (Cell Signaling; #14488; 1:1000) and beta-actin (Sigma-Aldrich; A5316; 1:5000). Antibodies were detected with IRDyes and scanned on the Odyssey CLx (LI-COR Biosciences). Relative band intensity was quantified using Odyssey Image Studio 2.0. All antibodies used in this study are listed in Table S2.

### Glucose-stimulated insulin secretion

Isolated mouse islets (∼50 islets per condition) were treated with 10 μM GR-46611 or vehicle control for 24 h and then starved in KREBS buffer (115 mM NaCl, 5 mM KCl, 24 mM NaHCO_3_, 2.5 mM CaCl_2_, 1 mM MgCl_2_, 10 mM Hepes, and 0.1% bovine serum albumin) supplemented with 2.8 mM glucose for 4 h. Islets were transferred to fresh KREBS buffer containing 2.8 mM glucose, and media were sampled after 30 min for basal secretion. Islets were then transferred to KREBS buffer containing 16.7 mM or 28 mM glucose and media were sampled after 30 min for glucose-stimulated insulin secretion. Islets were lysed in RIPA buffer containing protease and phosphatase inhibitor cocktail for total insulin content and insulin in lysates/media were measured by STELLUX Chemiluminescence Rodent Insulin ELISA (ALPCO). Data were calculated as % secreted insulin/total insulin and represented as average islet insulin secretion relative to vehicle basal.

### Statistical analysis

Statistical comparisons were performed using GraphPad Prism v10. Normality was assessed using the Shapiro–Wilk test, and homogeneity of variance was evaluated using the Brown–Forsythe test. If data met assumptions for parametric testing, unpaired, two-tailed Student’s t-tests or one-way/two-way ANOVA followed by *post hoc* multiple comparisons test were applied as appropriate. All data are shown as the mean ± standard deviations (SD). *p* values < 0.05 were considered significant (∗*p* < 0.05, ∗∗*p* < 0.01, ∗∗∗*p* < 0.001, ∗∗∗∗*p* < 0.0001). Details of sample size (n) and statistical analysis are provided in figure legends.

### Study approval

All research methods were approved by Stanford University’s Administrative Panel on Biosafety. Animal experiments were performed in compliance with IACUC protocols and approved by Stanford University’s Administrative Panel Laboratory Animal Care (APLAC). Human islet isolation was approved by the Human Research Ethics Board at the University of Alberta (Pro00013094). The studies in this work abide by the Declaration of Helsinki principles. All donors' families gave informed consent for the use of pancreatic tissue in research.

## Data availability

The data used to support the findings of this study are available from the corresponding author upon request.

## Supporting information

This article contains supporting information.

## Conflict of interest

The authors declare that they have no conflicts of interest with the contents of this article.
