# Supplementary material for: Pharmacologic stimulation of insulin granule acidification increases β-cell zinc content and augments β-cell-targeted drug delivery
Source: J Biol Chem. 2025 Aug 28;301(10):110645. doi: 10.1016/j.jbc.2025.110645 (PMC12494549; doi:10.1016/j.jbc.2025.110645)
Supplement: Supporting Figures and Tables [file mmc1.pdf]

SUPPORTING INFORMATION

Pharmacologic stimulation of insulin granule acidification increases β-cell zinc and augments β-cell-targeted drug delivery

Sooyeon Lee, Hannah P. Fraser, Rebecca C. Schugar, Haixia Xu, Timothy M. Horton, Ella A. Thomson, Julie Park, Xucheng Zhang, Justin P. Annes

Figure S1:

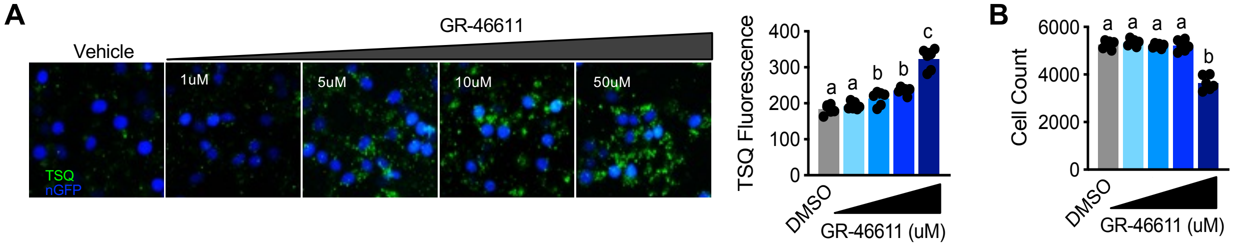

Figure S1. GR-46611 increases β-cell zinc. **A**, Representative live-imaging of TSQ fluorescence in R7T1:H2B-GFP β-cells treated 1, 5, 10 and 50 μM for 48h. Quantification of **B**, mean TSQ fluorescence intensity and **C**, cell counts, n=6/group

Figure S2:

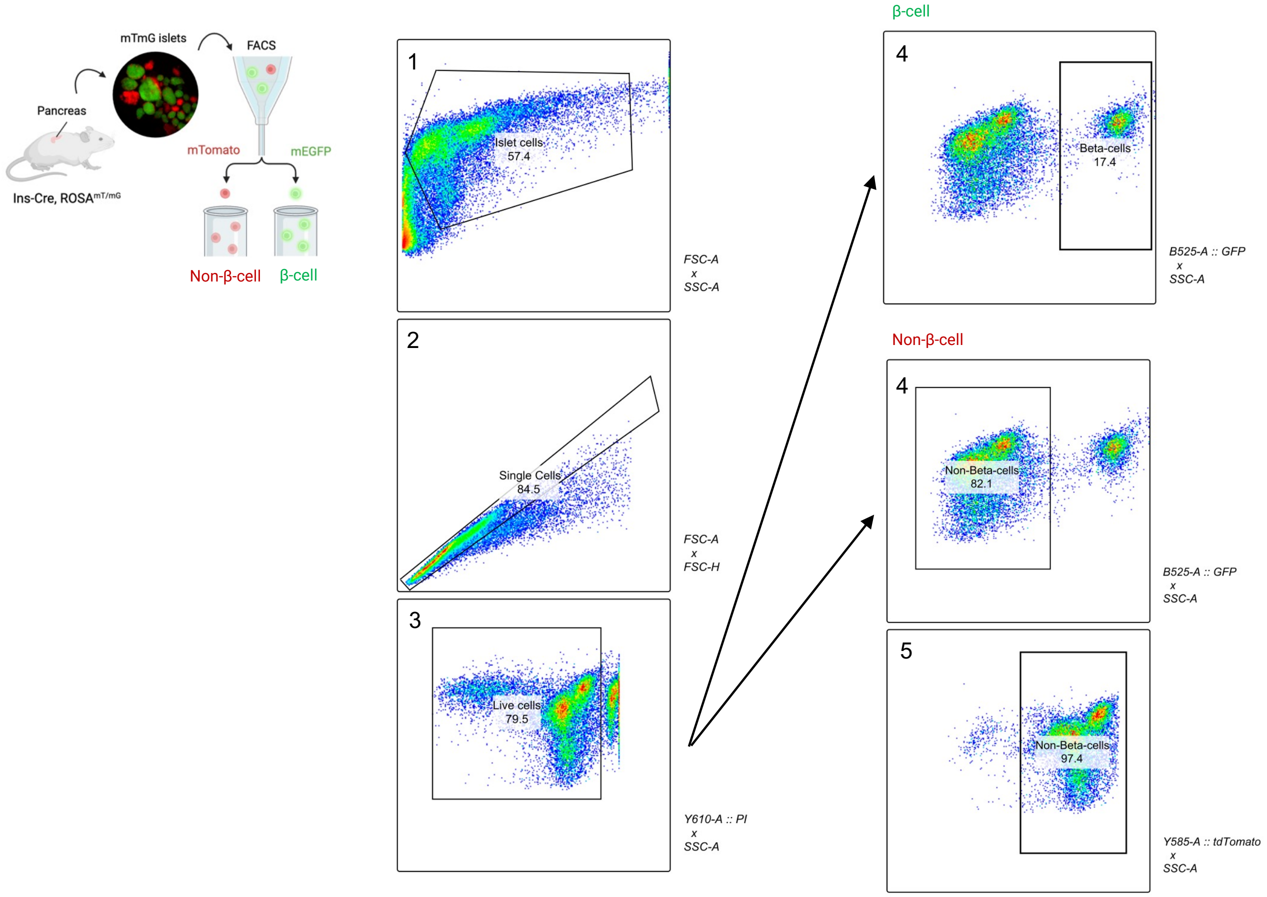

Figure S2. β-cell and non-β-cell sort from *mTmG* mouse islets. Gating scheme for sorting of β-cells and non-β-cells on BD LSRII.UV flow cytometer. Cell viability dye, propidium iodide (PI) was loaded for 15 min on ice to gate for PI-negative live cells. β-cells and non-β-cells were identified in B525 (GFP) and Y585 (tdTomato) channel, respectively.

Figure S3:

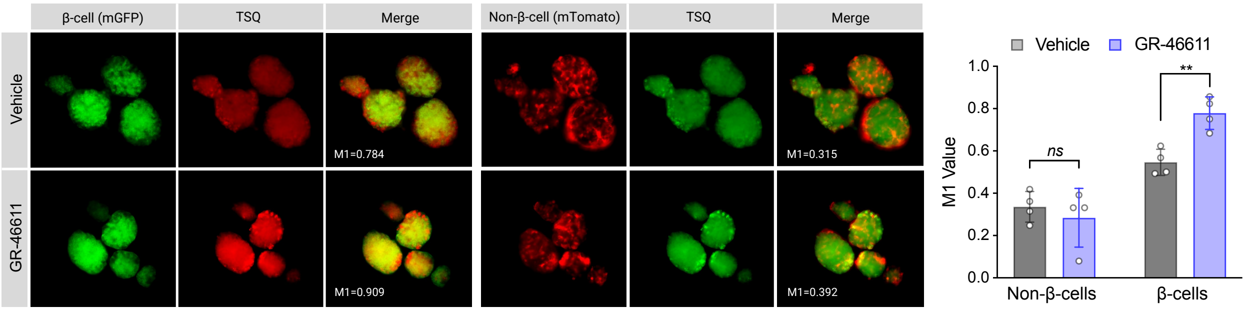

Figure S3. GR-46611 selectively increases zinc in β-cells of *mTmG* mouse islets. Representative live-imaging of isolated islets from dual-color fluorescent Cre-reporter mice (ROSA$^{mT/mG}$) crossed with insulin promoter-Cre recombinase (Ins2-Cre) mice (referred to as *mT/mG* mice). *mTmG* mouse islets express membrane-targeted green fluorescent protein (mGFP) in Cre-positive β-cells (green) and tandem dimer Tomato (mT) in Cre-negative non-β-cells (red). Islets were stained with TSQ, and fluorescence modified to red (left) or green (right) to demonstrate colocalized (merge) fluorescence in yellow. M1 Mander's coefficient – a measure of colocalization between two fluorescent signals are noted in images. Data represents mean M1 value in non-β-cells or β-cells treated with vehicle or GR-46611 for 4hr.

Figure S4:

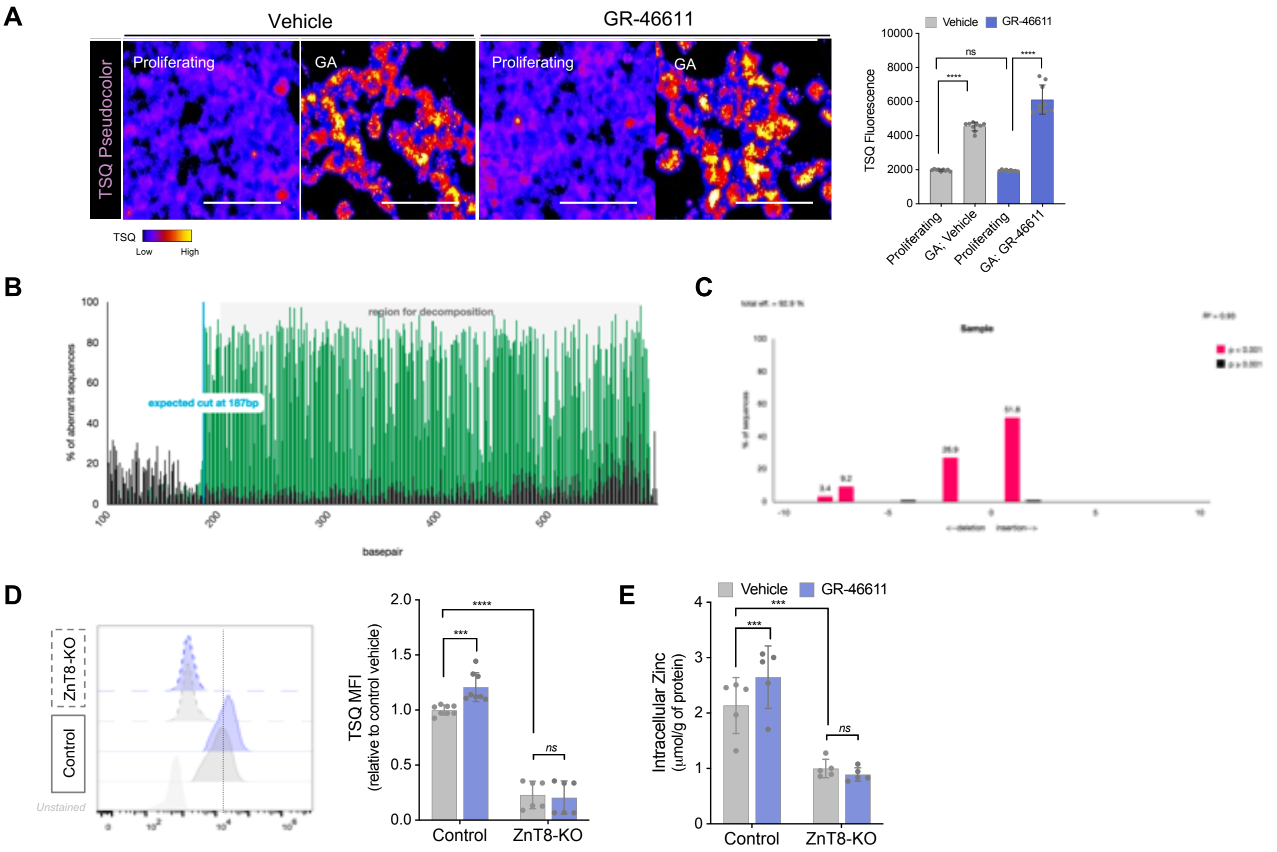

Figure S4. ZnT8-KO R7T1 β-cells fail to increase zinc upon GR-46611 treatment. A, Representative live-imaging of TSQ fluorescence following treatment with vehicle or 10 µM GR-46611 for 48 hr in proliferating vs growth-arrested R7T1 β-cells. TSQ shown as pseudocolor to demonstrate high and low changes in TSQ fluorescence intensity. Data represent mean TSQ fluorescence intensity (n=9/condition from three independent experiments). **B,** Analysis of gene editing efficiency by TIDE analysis. Representative plot of *slc30a8* (ZnT8) overlayed with control sample. The increase in aberrant sequence after the expected cut site is evident in the ZnT8-KO, indicating effective gene disruption. **C,** Graph represent the spectrum of indels and their frequencies for ZnT8. $R^2 = 0.93$. The plot was analyzed from the TIDE web tool (https://tide.nki.nl/). **D,** Histogram of TSQ fluorescence in control and ZnT8-KO R7T1 β-cells measured by flow cytometry. Data represents mean TSQ fluorescence intensity (MFI) relative to control vehicle. **E,** Zinc content measured in lysates from control and ZnT8-KO R7T1 β-cells (n= 5/condition).

Figure S5:

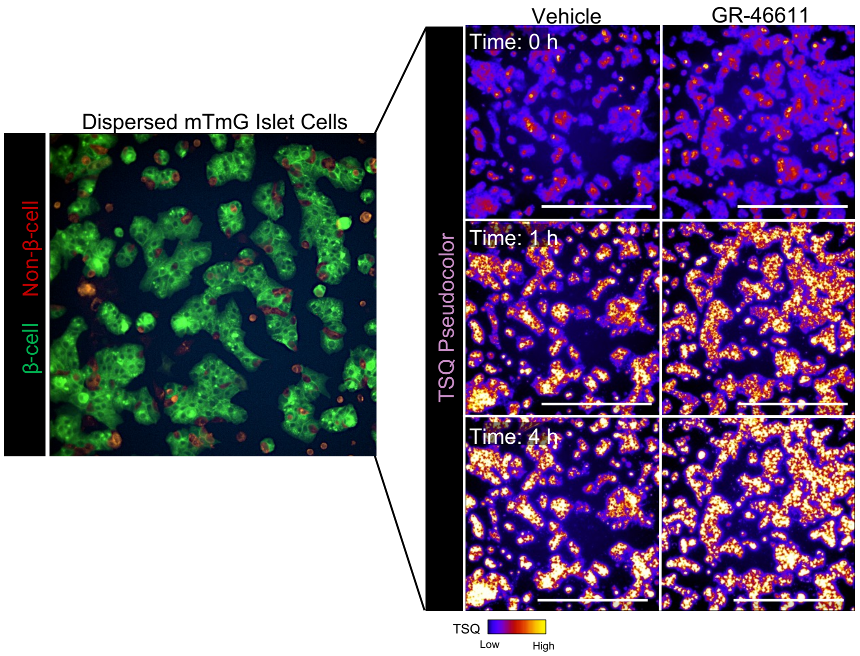

Figure S5. GR-46611 increases zinc flux in β-cells, but not non-β-cells.

Representative live-fluorescence images of dispersed mTmG islet cultures (left) and pseudocolored TSQ staining (right) at time 0, 1 and 4h of vehicle- or GR-46611treatments.

Figure S6:

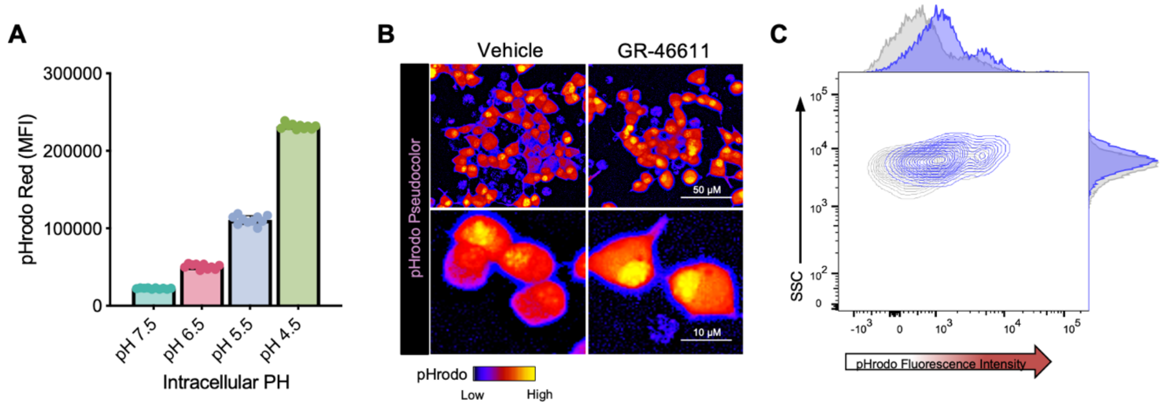

Figure S6. Intracellular acidification staining with pHrodo™.

A, pHrodo fluorescence quantification. Dispersed islet cells were loaded with pH calibration buffers (pH 4.5, 5.5, 6.5 or 7.5) containing 10 µM Valinomycin/Nigericin for 30 min. **B**, Representative pseudocolor pHrodo fluorescence images of live GA R7T1 β-cells treated with 10 µM GR-46611 or vehicle for 24 h. **C**, Representative FACS contour plots of pHrodo analysis in β-cells (mEGFP+) from mTmG mouse islets treated with vehicle or GR-46611 treatment for 4 h. x-axis , pHrodo fluorescence; y-axis, side scatter (SSC).

Figure S7:

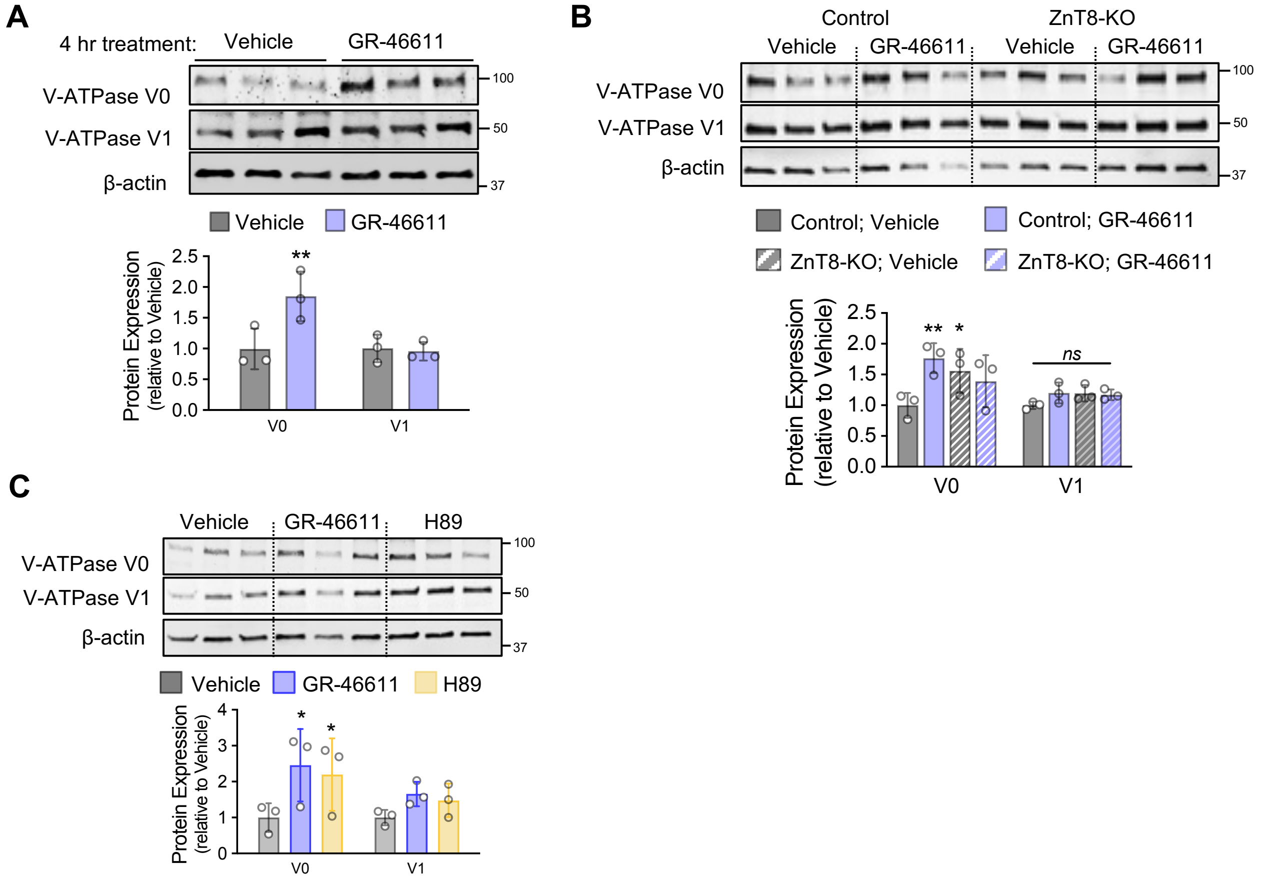

Figure S7. GR-46611 selectively increases expression of the V-ATPase V0 subunit without affecting the V1 subunit. A, Immunoblot of V-ATPase V0 and V1 in mouse islets treated with vehicle or 10 μM GR-46611 for 4 h. Graph represents β-actin-normalized protein expression relative to vehicle (n=3 mice). **B**, Immunoblot of V-ATPase V0 and V1 in Control and ZnT8-KO islets treated with vehicle or GR-46611 for 4 h. Graph represents β-actin-normalized protein expression relative to vehicle (n=3 mice/genotype). **C**, Immunoblot of V-ATPase V0 and V1 in mouse islets treated with vehicle, GR-46611 or H89 for 4 h. Graph represents β-actin-normalized protein expression relative to vehicle (n=3 mice). Data represent mean ± SD. Ordinary two-way ANOVA with Fisher's LSD post hoc test. $^{**}p < 0.01$, $^{*}p<0.05$, *ns*, non-significant.

Figure S8:

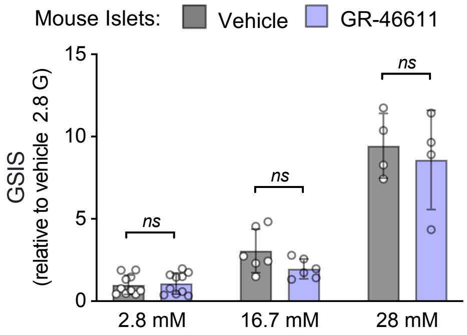

Figure S8. No effect of GR-46611 on glucose-stimulated insulin secretion.

Glucose-stimulated insulin secretion (GSIS) from mouse islets pre-treated with vehicle or 10 µM GR-46611 for 24h. Data represents GSIS (normalized to total protein concentration) relative to 2.8 mM glucose condition (*n*=4-10 mice).

Figure S9:

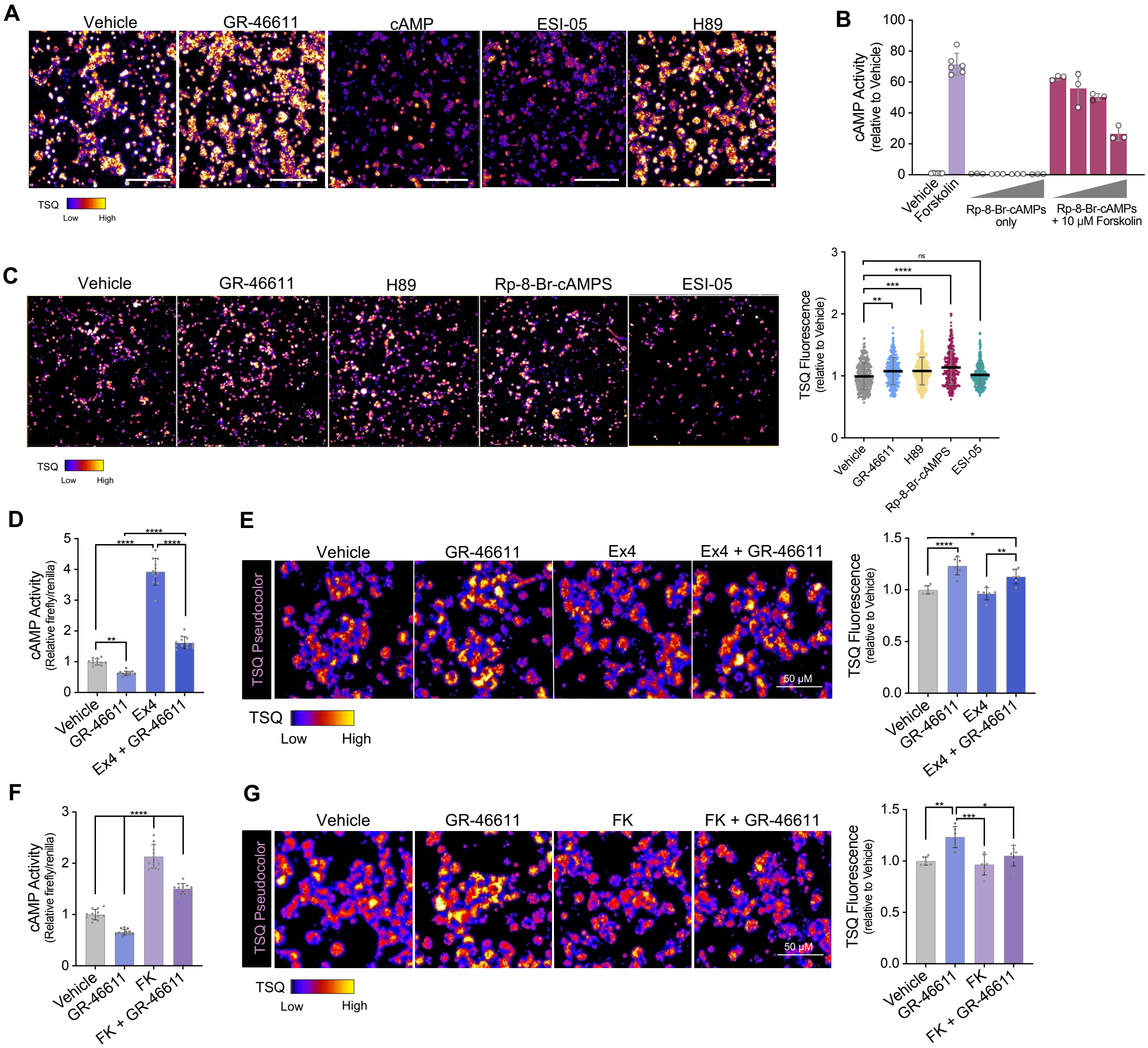

Figure S9. cAMP-PKA inhibition induces β-cell zinc accumulation.

A, Representative live-fluorescence images of pseudocolored TSQ staining in dispersed mTmG mouse islet cultures treated with compounds (10 µM) for 4h. **B**, Luciferase-based cAMP activity in HEK293 cells in response to increasing concentrations of Rp-8-Br-cAMPS (10, 50, 100, and 200 µM). Cells were either treated with Rp-8-Br-cAMPS alone or pre-treated for 4 hours prior to stimulation with forskolin. **C**, Representative pseudocolor TSQ fluorescence images showing intracellular zinc levels in live GA R7T1 β-cells treated with compounds (10 µM: GR-46611, H89, ESI-05; 100 µM: Rp-8-Br-cAMPS) for 48 h. **D, F** CRE-luciferase assay showing cAMP activity in GA R7T1 β-cells treated with vehicle, GR-46611, and/or Exendin-4 (Ex4, 10 nM, 48 h) (**D**) or Forskolin (FK, 10 nM, 48 h) (**F**). **E, G** Representative pseudocolor TSQ fluorescence images showing intracellular zinc levels in live GA R7T1 β-cells treated with Ex4 (**E**) or FK (**G**) for 48 h. Data represent mean ± SD. Ordinary two-way ANOVA with Fisher's LSD post hoc test. ****$p < 0.0001$, ***$p < 0.001$, **$p < 0.01$, *$p<0.05$, *ns*, non-significant.

Figure S10:

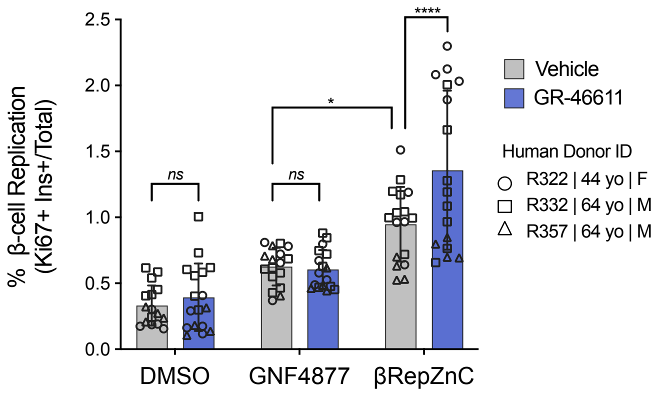

Figure S10. GR-46611 improves β-cell replication in human islets.

β-cell replication rate measured as percent of Ki67+ Insulin+ cells over total cell counts in human islets. Dispersed human islet cells were incubated with DMSO, GNF-4877(1.5 µM) or βRepZnC (1.5 µM) in combination with either vehicle or GR-46611 for 72 hr. Each symbol represents a different human donor. Donor ID, age and sex noted on graph.

Figure S11:

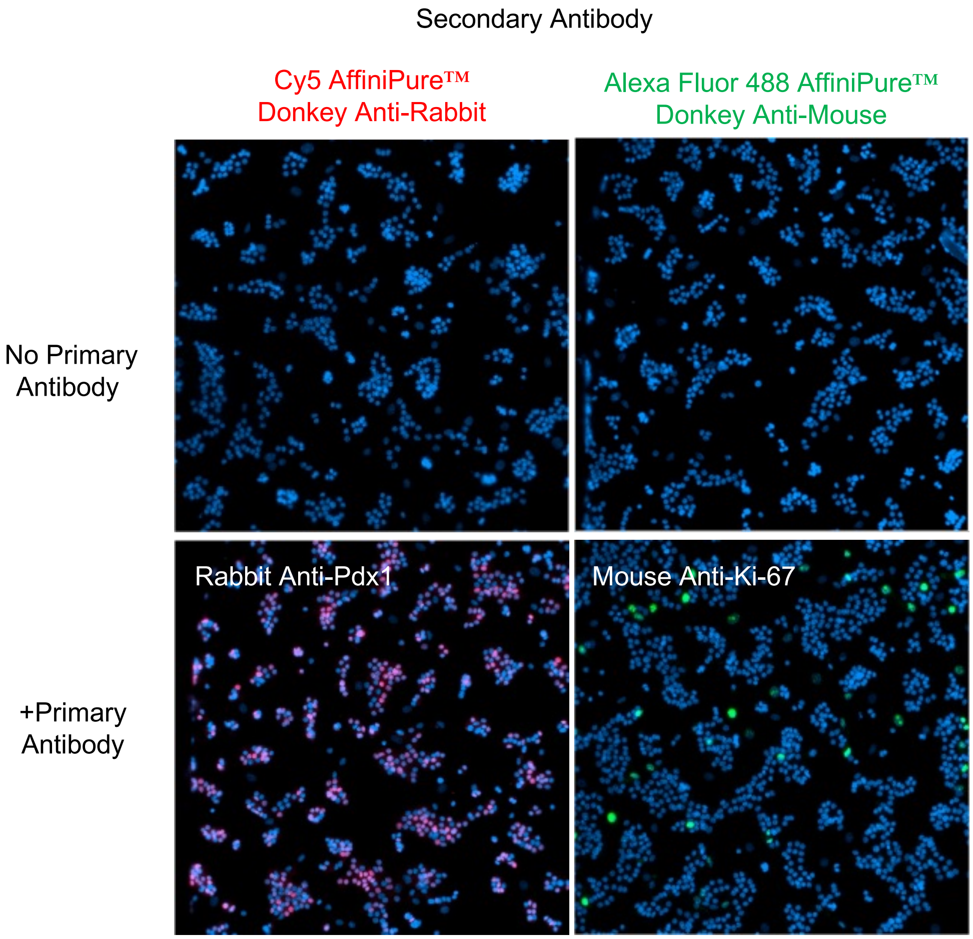

Figure S11. Negative staining control for secondary antibodies.
Dispersed islet cells stained with or without primary antibody incubation to assess nonspecific background signal from secondary antibodies: Cy5 and Alexa Fluor 488. No significant fluorescence signal was detected.

Table S1:

	Compound	Z-Score	Average TSQ relative to control	SD	% TSQ Δ
1	I-OMe-Tyrphostin AG 538	5.6	132.70	5.88	32.7
2	D-609 potassium	5.2	130.65	5.88	30.7
3	Perphenazine	4.9	139.65	8.16	39.6
4	2 3-Butanedione	4.8	146.14	9.52	46.1
5	1,10-Diaminodecane	4.6	143.88	9.52	43.9
6	(±) trans-U-50488 methanesulfonate	4.6	143.46	9.52	43.5
7	Naftopidil dihydrochloride	4.5	137.04	8.16	37.0
8	N^G N^G-Dimethylarginine hydrochloride	4.2	140.25	9.52	40.2
9	GR 46611	3.9	127.61	7.12	27.6
10	Budesonide	3.9	131.10	8.08	31.1
11	Ifenprodil tartrate	5.6	126.81	7.04	26.8
12	L-741,626	5.2	126.63	7.04	26.6
13	T-1032	4.9	121.56	5.88	21.6
14	Zardaverine	4.8	121.33	5.88	21.3
15	Bromoacetyl alprenolol menthane	4.6	128.19	8.14	28.2
16	LY-278584	4.6	122.84	7.04	22.8
17	PRE-084	4.5	124.83	8.16	24.8
18	Clorgyline hydrochloride	4.2	119.67	7.04	19.7
19	Tracazolate	3.9	114.52	5.88	14.5

Table S1. Zinc enhancers identified by TSQ-based chemical screening. Hit compounds identified from TSQ-based chemical screen of LOPAC 1280® compound library. Compounds with calculated z-scores > 2.5. were retested in replicates of 7 and confirmed as hits.

Table S2. Key Resources Table

REAGENT or RESOURCE	SOURCE	IDENTIFIER
**Antibodies**		
Monoclonal Mouse Anti-Ki-67	BD Pharmigen	550609
Monoclonal Rat Anti-BrdU	Abcam	Ab6326
Monoclonal Rabbit Anti-Pdx1	Cell Signaling Technology	5679
Monoclonal Rabbit Anti-Insulin	Cell Signaling Technology	3014
Monoclonal Rabbit Anti-ATP6V1B2	Cell Signaling Technology	14488
Polyclonal Rabbit Anti-ATP6V0A1	Sigma-Aldrich	SAB2108042
Monoclonal Mouse Anti-beta-Actin	Sigma-Aldrich	A5316
Alexa Fluor 488 AffiniPure™ Donkey Anti-Mouse IgG	Jackson ImmunoResearch	715-545-150
Alexa Fluor 488 AffiniPure™ Donkey Anti-Rat IgG	Jackson ImmunoResearch	712-545-150
Cy5 AffiniPure™ Donkey Anti-Rabbit IgG	Jackson ImmunoResearch	711-175-152
IRDye® 680RD Goat Anti-Mouse IgG	LI-COR Biosciences	925-68070
IRDye® 800CW Goat Anti-Rabbit IgG	LI-COR Biosciences	925-32211
**Bacterial and virus strains**		
Subcloning Efficiency™ DH5α Competent Cells	Thermo Fisher Scientific	18265017
**Biological samples**		
Human Pancreatic Islets	Alberta Diabetes Institute ADI Islet Core	R322, R332, R354, R357, R495, R511, R522
**Reagents, Chemicals, peptides, and recombinant proteins**		
DMEM High glucose with L-glutamine, sodium pyruvate	Cytiva	SH30243.01
DMEM Low glucose with L-glutamine, sodium pyruvate	Cytiva	SH30021.01
Fetal Bovine Serum	Cytiva	SH30910.03
Penicillin-Streptomycin	Cytiva	SV30010
Trypsin EDTA	Corning	25-053-CI
Doxycycline	Fisher Scientific	AC446060050
CIzyme	VitaCyte	005-1030
Histopaque®-1077	Sigma-Aldrich	10771
Histopaque®-1179	Sigma-Aldrich	11191
TSQ	MedChem Express	HY-119287
pHrodo™ Red	Thermo Fisher	P35372

Hoechst 33342	Thermo Scientific	62249
Propidium Iodide	Fisher Scientific	40017
LOPAC® 1280 compound library	Sigma-Aldrich	LO1280
Dimethyl Sulfoxide (DMSO)	Santa Cruz Biotechnology	sc-358801
GR-46611	Tocris	0864
Exendin-4	MedChem Express	HY-13443
Forskolin	MedChem Express	HY-15371
Bafilomycin A1	MedChem Express	HY-100558
H-89	MedChem Express	HY-15979
ESI-05	MedChem Express	HY-117656
8-Bromo-cAMP sodium salt	MedChem Express	HY-12306
TES sodium salt	Fisher Scientific	AAJ6270614
Mannitol	MP Biomedicals	02102248.5
Acetonitrile, Gradient Grade	Sigma-Aldrich	34851
Lipofectamine 2000	Thermo Fisher Scientific	11668019
Halt Protease & Phosphatase Inhibitor Cocktail	Thermo Fisher Scientific	78440
32% Paraformaldehyde	Electron Microscopy Sciences	15714-S
Formamide	Fisher Scientific	BP227
Intercept™ TBS Blocking Buffer	LI-COR Biosciences	927-60001
GNF-4877	Horton et al, 2019	N/A
4877-EXT-DPA (βRepZnC)	Horton et al, 2019	N/A
4877-EXT-DBA (βRepNC)	Horton et al, 2019	N/A
**Critical commercial assays**		
Stellux® Chemi Rodent Insulin ELISA Jumbo	Alpco	80-INSMR-CH10
Pierce™ BCA Protein Assay Kits	Thermo Scientific	23227
**Experimental models: Cell lines**		
R7T1	Milo-Landesman et al., 2001	RRID:CVCL_AW02
HEK293T	ATCC	CRL-3216
**Experimental models: Organisms/strains**		
C57BL/6J mice	The Jackson Laboratory	000664
B6.Cg-Tg(Ins2-cre)25Mgn/J	The Jackson Laboratory	003573
B6.129(Cg)-*Gt(ROSA)26Sor*$^{tm4(ACTB-tdTomato,-EGFP)Luo}$/J (mTmG) mice	The Jackson Laboratory	007676
Slc30a8$^{tm1a(KOMP)Wtsi}$ (ZnT8KO)	Syring et al, 2020	MGI:4363904
Sprague-Dawley rats	Charles River	001

**Oligonucleotides**		
**Recombinant DNA**		
Plasmid: H2B-GFP	Addgene	11680
Plasmid: pRSV-Rev	Addgene	12253
Plasmid: pMDLg/pRRE	Addgene	12251
Plasmid: pMD2.G	Addgene	12259
Plasmid: lentiCas9-Blast	Addgene	52962
Plasmid: pMCB320	Addgene	89359
Plasmid: pGL4.29[luc2P/CRE/Hygro]	Promega	E847A
Plasmid: pRL-CMV	Promega	E2261

**sgRNA Sequence**	
Slc30A8 (ZnT8)	AAGATCAGTGTCCTGGAGAC
Ins1	TGTGGATGCGCTTCCTGCCC
Ins2	GCTCTTCCTCTGGGAGTCCC

**Software and algorithms**		
GraphPad Prism 10	GraphPad	http://www.graphpad.com/
FlowJo 10.10	BD Biosciences	https://www.flowjo.com/
Harmony®	Revvity	https://www.revvity.com/category/cell-analysis
Cellomics™	Thermo Fisher Scientific	https://www.thermofisher.com/us/en/home/brands/thermo-scientific/cellomics.html
Odyssey Image Studio™	LI-COR Biosciences	https://www.licor.com/bio/image-studio/
**Other**		
ArrayScan VTI HCS	Thermo Fisher	N/A
SpectraMax iD3 plate reader	Molecular Devices	N/A
Operetta CLS	Perkin Elmer	N/A

Supplemental Video Legends

Video S1. Time-lapse of mTmG islets treated with DMSO (vehicle), loaded with TSQ (blue), and monitored over a 4 hr period. β-cells in green; non-β-cells in red. 1 time frame per sec.

Video S2. Time-lapse of mTmG islets treated with 10 μM GR-46611, loaded with TSQ (blue), and monitored over a 4 hr period. β-cells in green; non-β-cells in red. 1 time frame per sec.

Video S3. Time-lapse of mTmG islets treated with DMSO (vehicle), loaded with TSQ (blue), and monitored over a 4 hr period. β-cells in green. 1 time frame per sec; total 50 frames.

Video S4. Time-lapse of mTmG islets treated with 10 μM GR-46611, loaded with TSQ (blue), and monitored over a 4 hr period. β-cells in green. 1 time frame per sec; total 50 frames.

Video S5. Time-lapse of mTmG islets co-treated with 10 μM GR-46611 and 100 nM Bafilomycin A1, loaded with TSQ (blue), and monitored over a 4 hr period. β-cells in green. 1 time frame per sec; total 50 frames.

Video S6. Time-lapse of mTmG islets treated with DMSO (vehicle), loaded with TSQ (blue), and monitored over a 4 hr period. β-cells in green. 1 time frame per sec; total 50 frames.

Video S7. Time-lapse of mTmG islets treated with 10 μM GR-46611, loaded with TSQ (blue), and monitored over a 4 hr period. β-cells in green. 1 time frame per sec; total 50 frames.

Video S8. Time-lapse of mTmG islets treated with 10 μM cAMP, loaded with TSQ (blue), and monitored over a 4 hr period. β-cells in green. 1 time frame per sec; total 50 frames.

Video S9. Time-lapse of mTmG islets treated with 10 μM ESI-05, loaded with TSQ (blue), and monitored over a 4 hr period. β-cells in green. 1 time frame per sec; total 50 frames.

Video S10. Time-lapse of mTmG islets treated with 10 μM H89, loaded with TSQ (blue), and monitored over a 4 hr period. β-cells in green. 1 time frame per sec; total 50 frames.
